# Men missing from the HIV care continuum in sub‐Saharan Africa: a meta‐analysis and meta‐synthesis

**DOI:** 10.1002/jia2.25889

**Published:** 2022-03-24

**Authors:** Maria F. Nardell, Oluwatomi Adeoti, Carson Peters, Bernard Kakuhikire, Caroline Govathson‐Mandimika, Lawrence Long, Sophie Pascoe, Alexander C. Tsai, Ingrid T. Katz

**Affiliations:** ^1^ Department of Medicine Brigham and Women's Hospital Boston Massachusetts USA; ^2^ Department of Medicine Beth Israel Deaconess Medical Center Boston Massachusetts USA; ^3^ Harvard Medical School Boston Massachusetts USA; ^4^ Department of Medicine Boston Medical Center Boston Massachusetts USA; ^5^ Department of Epidemiology University of Iowa College of Public Health Iowa City Iowa USA; ^6^ Faculty of Business and Management Sciences Mbarara University of Science and Technology Mbarara Uganda; ^7^ Health Economics and Epidemiology Research Office University of the Witwatersrand Johannesburg South Africa; ^8^ Department of Internal Medicine School of Clinical Medicine University of the Witwatersrand Johannesburg South Africa; ^9^ Department of Global Health Boston University School of Public Health Boston Massachusetts USA; ^10^ Mongan Institute Massachusetts General Hospital Boston Massachusetts USA; ^11^ Center for Global Health Massachusetts General Hospital Boston Massachusetts USA; ^12^ Harvard Global Health Institute Cambridge Massachusetts USA

**Keywords:** Africa South of the Sahara, continuity of patient care, HIV infections, HIV testing, men, qualitative research

## Abstract

**Introduction:**

Men are missing along the HIV care continuum. However, the estimated proportions of men in sub‐Saharan Africa meeting the UNAIDS 95‐95‐95 goals vary substantially between studies. We sought to estimate proportions of men meeting each of the 95‐95‐95 goals across studies in sub‐Saharan Africa, describe heterogeneity, and summarize qualitative evidence on factors influencing care engagement.

**Methods:**

We systematically searched PubMed and Embase for peer‐reviewed articles published between 1 January 2014 and 16 October 2020. We included studies involving men ≥15 years old, with data from 2009 onward, reporting on at least one 95‐95‐95 goal in sub‐Saharan Africa. We estimated pooled proportions of men meeting these goals using DerSimonion‐Laird random effects models, stratifying by study population (e.g. studies focusing exclusively on men who have sex with men vs. studies that did not), facility setting (healthcare vs. community site), region (eastern/southern Africa vs. western/central Africa), outcome measurement (e.g. threshold for viral load suppression), median year of data collection (before vs. during or after 2017) and quality criteria. Data from qualitative studies exploring barriers to men's HIV care engagement were summarized using meta‐synthesis.

**Results and discussion:**

We screened 14,896 studies and included 129 studies in the meta‐analysis, compiling data over the data collection period. Forty‐seven studies reported data on knowledge of serostatus, 43 studies reported on antiretroviral therapy use and 74 studies reported on viral suppression. Approximately half of men with HIV reported not knowing their status (0.49 [95% CI, 0.41–0.58; range, 0.09–0.97]) or not being on treatment (0.58 [95% CI, 0.51–0.65; range, 0.07–0.97]), while over three‐quarters of men achieved viral suppression on treatment (0.79 [95% CI, 0.77–0.81; range, 0.39–0.97]. Heterogeneity was high, with variation in estimates across study populations, settings and outcomes. The meta‐synthesis of 40 studies identified three primary domains in which men described risks associated with engagement in HIV care: perceived social norms, health system challenges and poverty.

**Conclusions:**

Psychosocial and systems‐level interventions that change men's perceptions of social norms, improve trust in and accessibility of the health system, and address costs of accessing care are needed to better engage men, especially in HIV testing and treatment.

## 1 INTRODUCTION

Men are disproportionately missing, compared with women, throughout the HIV care continuum in sub‐Saharan Africa [1, 2, 3] and have higher mortality from HIV‐related illnesses [2, 4, 5]. This gap must be bridged if UNAIDS 95‐95‐95 fast‐track goals are to be achieved by 2030 – so that 95% of persons with HIV (PWH) know their status, 95% of persons with known HIV are on antiretroviral therapy (ART) and 95% of PWH on ART are virally suppressed [6]. UNAIDS 2020 estimates show substantial heterogeneity in achieving these goals across the continent, with higher proportions of men meeting these goals in eastern and southern Africa as compared to western and central Africa [5]. However, national and regional data do not capture variability across subgroups of men, which would help target resources towards those who need it most. Moreover, while national programs provide updated yearly data on these goals, examining data over an extended period provides a more nuanced understanding of where there have been, and may continue to be, gaps over time despite some yearly gains, particularly for certain highly vulnerable populations.

Efforts to effectively engage men in HIV care must be informed not only by estimates of where and how they experience challenges along the care continuum but also by a better understanding of subgroup variation. Recent work on the UNAIDS goals has documented socio‐demographic heterogeneity among men who have sex with men (MSM) [7] and among both men and women [8], but the latter study was limited by the availability of data. Certain groups of men are at higher risk of being missed by HIV care, including older men [9] and MSM [7]. However, men are often treated as a homogenous population in assessments of progress towards the UNAIDS goals, without disaggregation by socio‐demographic factors, including education, employment and mobility [10]. There are also challenges in consistently estimating UNAIDS goals due to variation in how they are measured [9, 11]. The extent to which this variation may affect population‐level estimates of men's progression along the continuum is unknown.

Care‐seeking decisions may be contextualized within the framework of risk perception, drawn from behavioural economics, which suggests that people are highly loss averse, meaning that they generally prefer to avoid losses more than they prefer an equivalent gain [12, 13]. This framework has been used to understand HIV care engagement [14] in showing that people are highly influenced by subjective concerns (ie, “losses” or risks), including stigma and costs, which can discourage seeking care [14]. However, it is unclear which perceived risks of HIV care engagement are most salient for men across different settings in sub‐Saharan Africa. This is an important gap in the literature because such information may help guide the design of scalable interventions. While strategies have been designed to engage men in HIV care, including community‐based programs, workplace testing and comprehensive men's health services, data remain limited on their effectiveness [15, 16].

To address these gaps, we conducted a meta‐analysis to estimate the pooled proportion of men in sub‐Saharan Africa meeting the 95‐95‐95 goals and to describe heterogeneity across studies in sub‐Saharan Africa with the aim of identifying which subgroups of men may be most vulnerable throughout the continuum. We applied meta‐synthesis to qualitative studies on factors influencing men's engagement in HIV care to elucidate potential psychosocial and structural drivers of our quantitative findings and identify avenues for intervention.

## 2 METHODS

### 2.1 Search strategy and selection criteria

We systematically searched PubMed and Embase for peer‐reviewed articles published after 1 January 2014 (the year in which the UNAIDS goals were set) for consideration of the meta‐analysis or meta‐synthesis (Appendix). For the meta‐analysis, we included cross‐sectional, longitudinal, case–control or randomized trial (including only the control arm) studies conducted in sub‐Saharan Africa involving men ≥15 years of age in which at least part of the sample was enrolled on or after 1 January 2009, so as to focus on the modern HIV testing and treatment era. If studies with data after 2009 included data spanning years prior to 2009, they were included. For the meta‐synthesis, we included qualitative or mixed method studies conducted in sub‐Saharan Africa exploring factors influencing men's engagement in any stage of the continuum, enrolling participants on or after 1 January 2009. For the meta‐analysis and meta‐synthesis, we excluded mathematical modelling studies or studies lacking data disaggregated by sex. The evidence searches were conducted on 15 July 2019. We updated the searches to identify additional studies for the meta‐synthesis on 1 July 2020 and to identify additional studies for the meta‐analysis on 16 October 2020.

We imported all records into Covidence systematic review management software, automatically excluding duplicates [17]. We screened titles and abstracts and then screened the remaining full manuscripts to select studies meeting inclusion criteria for the meta‐analysis and/or meta‐synthesis. Conflicts between any two reviewers were resolved through discussion with a third reviewer.

For the meta‐analysis, we independently extracted the following primary outcomes of interest, selected *a priori*: the numerator and denominator of men meeting any 95‐95‐95 goal(s) reported. For studies that reported sex‐disaggregated data, we extracted the numerator and denominator of women meeting any 95‐95‐95 goal(s) reported. For the first 95‐95‐95 goal, the numerator was defined as “persons with HIV aware of their serostatus,” and the denominator was defined as “persons with HIV.” For the second 95‐95‐95 goal, the numerator was defined as “persons with HIV on antiretroviral therapy,” and the denominator was defined as “persons with HIV aware of their serostatus.” For the third 95‐95‐95 goal, the numerator was defined as “persons with HIV on antiretroviral therapy and virally suppressed,” and the denominator was defined as “persons with HIV on antiretroviral therapy” (Table A1). In publications where data were not disaggregated by sex, we emailed study authors to request sex‐specific estimates. We extracted data on study and population characteristics for each 95‐95‐95 goal. Study characteristics included: country, setting (rural vs. urban), facility (healthcare vs. community‐based), year of publication and study period year(s). Population characteristics included: employment status, occupation, migratory status, relationship status, sexual minority status (exclusively focused on MSM vs. not exclusively focused on MSM), HIV prevalence as documented in the study data and age of participants.

To assess variation in how the 95‐95‐95 goals were measured, we extracted the following data: whether knowledge of serostatus was ascertained pre‐ versus post‐testing campaign, whether ART status was measured by self‐report or blood test, and the viral load threshold and minimum follow‐up time on ART when viral suppression was ascertained.

To identify items that should be included in our quality assessment of the quantitative studies, we referenced the Newcastle‐Ottawa Quality Assessment Scale for observational studies and the Revised Cochrane risk‐of‐bias tool for randomized trials [18, 19]. To make our quality review straightforward to implement among multiple reviewers, we focused on items most relevant to our analyses of the 95‐95‐95 goals, including the sampling and recruitment process as well as setting, participant characteristics and goal measurement. Therefore, we inspected the full text of manuscripts for clear descriptions of (1) the study setting; (2) the participant selection process; (3) participant characteristics; and (4) the measurement of the 95‐95‐95 goal(s). We categorized the studies into two quality categories: “all criteria met” or, if any of the four criteria were not met, “criteria partially met.” Quality assessment for the qualitative studies was based on criteria used in prior research [20, 21], representing the key conceptual domains in the Critical Appraisal Skills Programme quality assessment tool [22]: clear descriptions of (1) the role of the researcher; (2) the sampling method; (3) the method of data collection; and (4) the method of analysis. Again, we categorized the studies into two quality categories: “all criteria met” or “criteria partially met.”

MFN, OA and CP independently conducted all stages of screening and data extraction. All data were cross‐checked and discrepancies were resolved by consensus.

### 2.2 Data analysis

Using Stata statistical software (version 16, StataCorp LLC, College Station, TX), we transformed proportions using the Freeman–Tukey variance‐stabilizing double arcsine transformation [23]. We computed pooled estimates of prevalence using the DerSimonian and Laird random effects model [24]. Study‐specific confidence intervals were estimated using the score method [25, 26]. We characterized the extent of heterogeneity between studies using the *I* ^2^ statistic [27]. We re‐estimated pooled prevalence stratified by available study‐level variables. The systematic review and meta‐analysis were reported in accordance with PRISMA guidelines [28].

For qualitative studies, we used the iterative process of meta‐synthesis, which stems from early methodology proposed by Noblit and Hare [29] and has come to define a collection of approaches for synthesizing qualitative research [30, 31]. Our process of meta‐synthesis is adapted from more recent interpretations, including approaches used in thematic synthesis [21, 32]. We summarized key themes from the studies, which formed the basis of second‐order constructs, defined as the study authors’ interpretations of participants’ beliefs. We resolved discrepancies through team discussion and created a codebook of second‐order constructs and first‐order constructs, that is direct quotations from study participants. We generated a summary definition for each second‐order construct, which was consolidated into a line of argument leading to a third‐order analysis. We grouped third‐order constructs into broad third‐order labels encompassing domains in which men described perceived risks of engagement in HIV care. Based on participant quotations, we identified factors that heightened men's perceived risks of engagement in care (“barriers”) and factors that lessened their perceived risk and facilitated initial engagement in care and/or reinforced ongoing engagement. While these “facilitators” of care did not address all barriers that men face, we grouped them under the third‐order labels to highlight where there may be potential in mitigating some perceived risks of engagement.

## 3 RESULTS AND DISCUSSION

**Figure 1:**
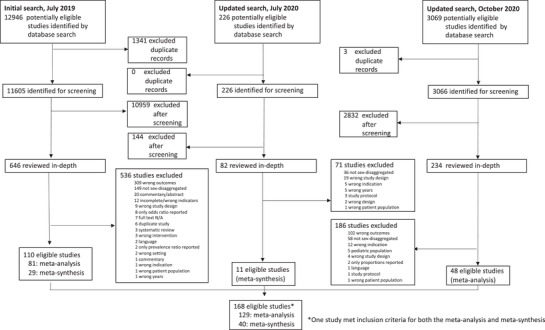
Study selection. Search process for selected studies in meta‐analysis and meta‐synthesis.

Our initial search identified 12,946 articles for screening, of which 1341 were removed as duplicates (Figure 1). We screened titles and abstracts of the remaining 11,605 studies, excluding 10,959 records that did not meet inclusion criteria, and reviewed the full text of 646 articles. Of these, 81 studies were included in the meta‐analysis [33, 34, 35, 36, 37, 38, 39, 40, 41, 42, 43, 44, 45, 46, 47, 48, 49, 50, 51, 52, 53, 54, 55, 56, 57, 58, 59, 60, 61, 62, 63, 64, 65, 66, 67, 68, 69, 70, 71, 72, 73, 74, 75, 76, 77, 78, 79, 80, 81, 82, 83, 84, 85, 86, 87, 88, 89, 90, 91, 92, 93, 94, 95, 96, 97, 98, 99, 100, 101, 102, 103, 104, 105, 106, 107, 108, 109, 110, 111, 112, 113] and 29 studies were included in the meta‐synthesis [105, 114, 115, 116, 117, 118, 119, 120, 121, 122, 123, 124, 125, 126, 127, 128, 129, 130, 131, 132, 133, 134, 135, 136, 137, 138, 139, 140, 141]. Our updated searches identified 48 additional studies for the meta‐analysis [142, 143, 144, 145, 146, 147, 148, 149, 150, 151, 152, 153, 154, 155, 156, 157, 158, 159, 160, 161, 162, 163, 164, 165, 166, 167, 168, 169, 170, 171, 172, 173, 174, 175, 176, 177, 178, 179, 180, 181, 182, 183, 184, 185, 186, 187, 188, 189] and 11 additional studies for the meta‐synthesis [190, 191, 192, 193, 194, 195, 196, 197, 198, 199, 200], most published in 2020.

### 3.1 Meta‐analysis

For the meta‐analysis, 47 studies reported data on knowledge of positive serostatus [39, 41, 44, 52, 53, 54, 55, 58, 59, 63, 64, 65, 69, 73, 77, 79, 80, 81, 83, 84, 86, 91, 94, 95, 97, 99, 101, 102, 108, 110, 111, 112, 113, 144, 146, 149, 151, 154, 157, 167, 171, 178, 185, 186, 188, 189], 43 studies reported data on ART use [35, 36, 39, 47, 51, 55, 60, 62, 67, 70, 83, 84, 85, 95, 97, 98, 99, 104, 105, 107, 108, 112, 142, 144, 145, 146, 148, 149, 150, 154, 161, 162, 166, 167, 168, 170, 171, 181, 184, 185, 186, 188, 189] and 74 studies reported data on viral suppression (Table 1 and Table A2) [33, 34, 37, 38, 40, 41, 42, 43, 45, 46, 48, 49, 50, 55, 56, 57, 61, 66, 68, 71, 72, 74, 75, 76, 78, 82, 84, 87, 88, 89, 90, 92, 93, 95, 96, 97, 100, 103, 106, 107, 108, 109, 143, 146, 147, 149, 152, 153, 154, 155, 156, 158, 159, 160, 161, 163, 164, 165, 169, 171, 172, 173, 174, 175, 176, 177, 179, 180, 182, 183, 185, 186, 187, 188]. While all studies included data collected in 2009 or later, some studies included data spanning back to 2002 and as recent as 2019, representing 1,564,019 participants in 21 countries. South Africa was the most represented country (40 [31.0%]). Three studies included representation from eastern and southern Africa as well as western and central Africa [76, 150, 180]; of the remaining studies, eastern and southern Africa was more represented (113 [89.7%]) as compared to western and central Africa (13 [10.3%]). The median number of participants was 1688 (interquartile range [IQR], 552–5666; range, 63–248,002). Studies reporting data on knowledge of positive status were most often conducted in community settings (31/47 [66.0%]), as were studies reporting data on ART status (27/43 [62.8%]). In contrast, most studies reporting data on viral suppression were conducted in healthcare facilities (55/74 [74.3%]). MSM were the focus of 14 studies [47, 53, 58, 64, 65, 67, 78, 80, 84, 91, 97, 144, 154, 186]. Nearly, half of studies (61/129 [47.3%]) only partially met quality criteria.

**Table 1:** Characteristics of studies included in meta‐analysis (*N* = 129)

Characteristics	Studies (*n*, %)
Study design	
Prospective cohort	33 (25.6)
Retrospective cohort	24 (18.6)
Cross‐sectional	63 (48.8)
Case–control	2 (1.6)
Randomized trial^a^	7 (5.4)
Population focus	
MSM	14 (10.9)
Heterosexual men or not specified	115 (89.1)
Transgender women	5 (3.9)
Transgender men (explicitly included)	1 (0.8)
Migrant men	1 (0.8)
Year of publication	
2014–2016	31 (24.0)
2017–2018	34 (26.4)
2019–2020	64 (49.6)
Region/country^b^	
Eastern and southern Africa^c^, ^d^	113 (89.7)
South Africa	39 (31.0)
Kenya	19 (15.1)
Uganda	18 (14.3)
Other	Angola (1), Botswana (4), Ethiopia (7), Lesotho (1), Malawi (5), Mozambique (3), Rwanda (6), Swaziland (2), United Republic of Tanzania (6), Zambia (9), Zimbabwe (4)
Western and central Africa^e^, ^f^	13 (10.3)
Nigeria	7 (5.6)
Other	Burkina Faso (1), Cameroon (2), Ghana (1), Mali (1), Senegal (1), Togo (1)
Quality criteria	
All criteria met	68 (52.7)
Criteria partially met	61 (47.3)

^a^ Data from randomized trials obtained from control arm.

^b^ *N* = 126 because three studies included compiled data from countries in both regions.

^c^ Angola, Botswana, Comoros, Eritrea, Eswatini, Ethiopia, Kenya, Lesotho, Madagascar, Malawi, Mauritius, Mozambique, Namibia, Rwanda, Seychelles, South Africa, South Sudan, Uganda, United Republic of Tanzania, Zambia and Zimbabwe.

^d^ There are nine studies representing eastern and southern Africa that include more than one country from this region.

^e^ Benin, Burkina Faso, Burundi, Cabo Verde, Cameroon, Central African Republic, Chad, Congo, Cote d'Ivoire, Democratic Republic of Congo, Equatorial Guinea, Gabon, Gambia, Ghana, Guinea, Guinea‐Bissau, Liberia, Mali, Mauritania, Niger, Nigeria, Sao Tome and Principe, Senegal, Sierra Leone and Togo.

^f^ There is one study representing western and central Africa that includes more than one country from this region.

Most studies reporting on knowledge of positive status asked participants about their status prior to testing in the study (33/47 [70.2%]), whereas some studies provided unclear details (7/47 [14.9%]) or used other methods (7/47 [14.9%]), including asking about knowledge of status after testing within the study. Most studies measured treatment status by self‐report (23/43 [53.5%]), whereas seven studies used a blood test for ART detection (16.3%), five studies used chart documentation (11.6%) and eight studies used more than one method (18.6%). Most studies reporting on viral suppression used 1000 copies/ml as the threshold detection limit of viral suppression (44 [59.5%]), but the limit ranged from 20 to 5000 copies/ml. The minimum amount of time on ART required for measuring viral load varied from 2 to 24 months.

**Figure 2:**
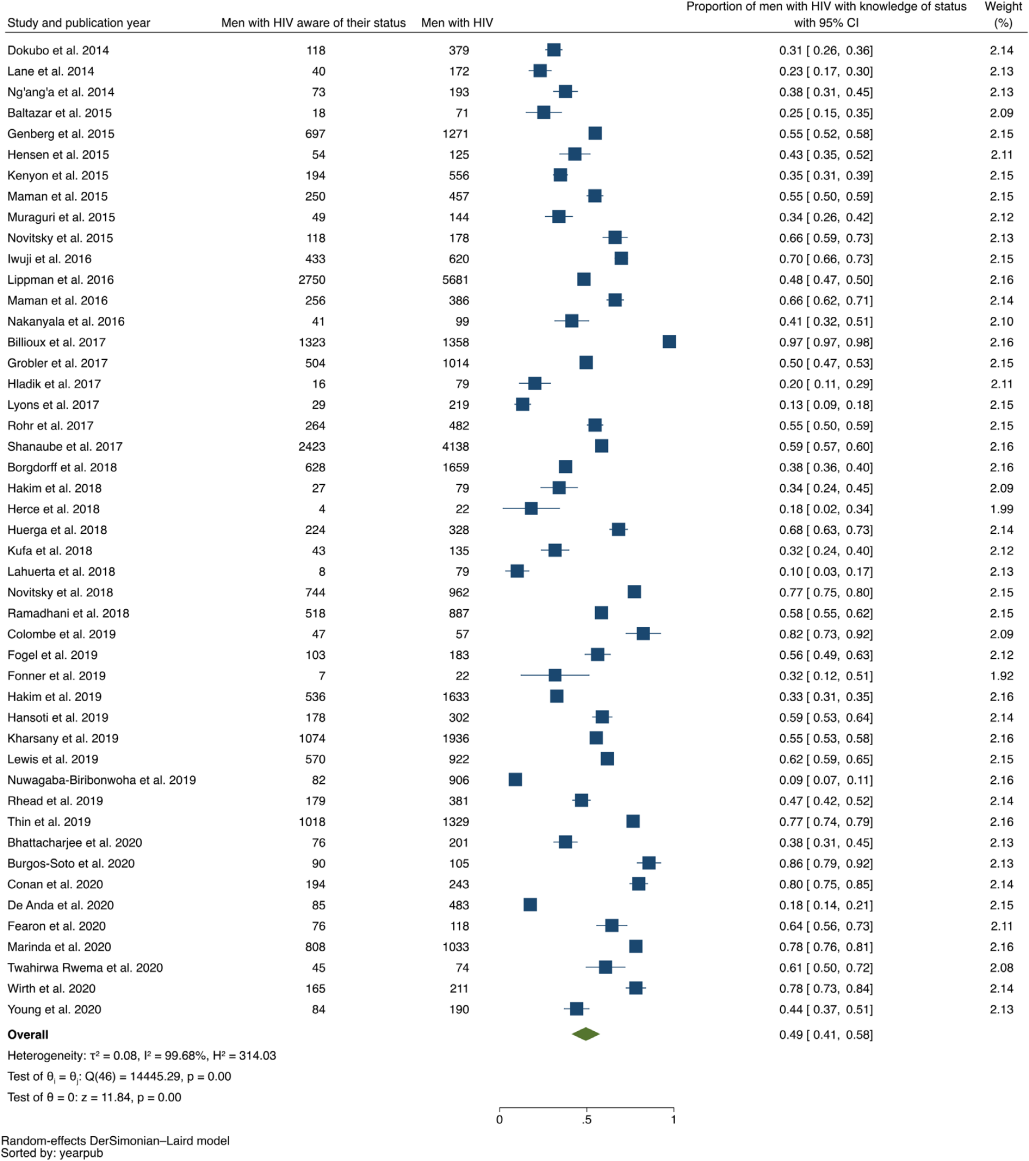
Forest plot of studies reporting data on proportion of men with HIV with knowledge of their status, listed in ascending order of year of publication.

**Figure 3:**
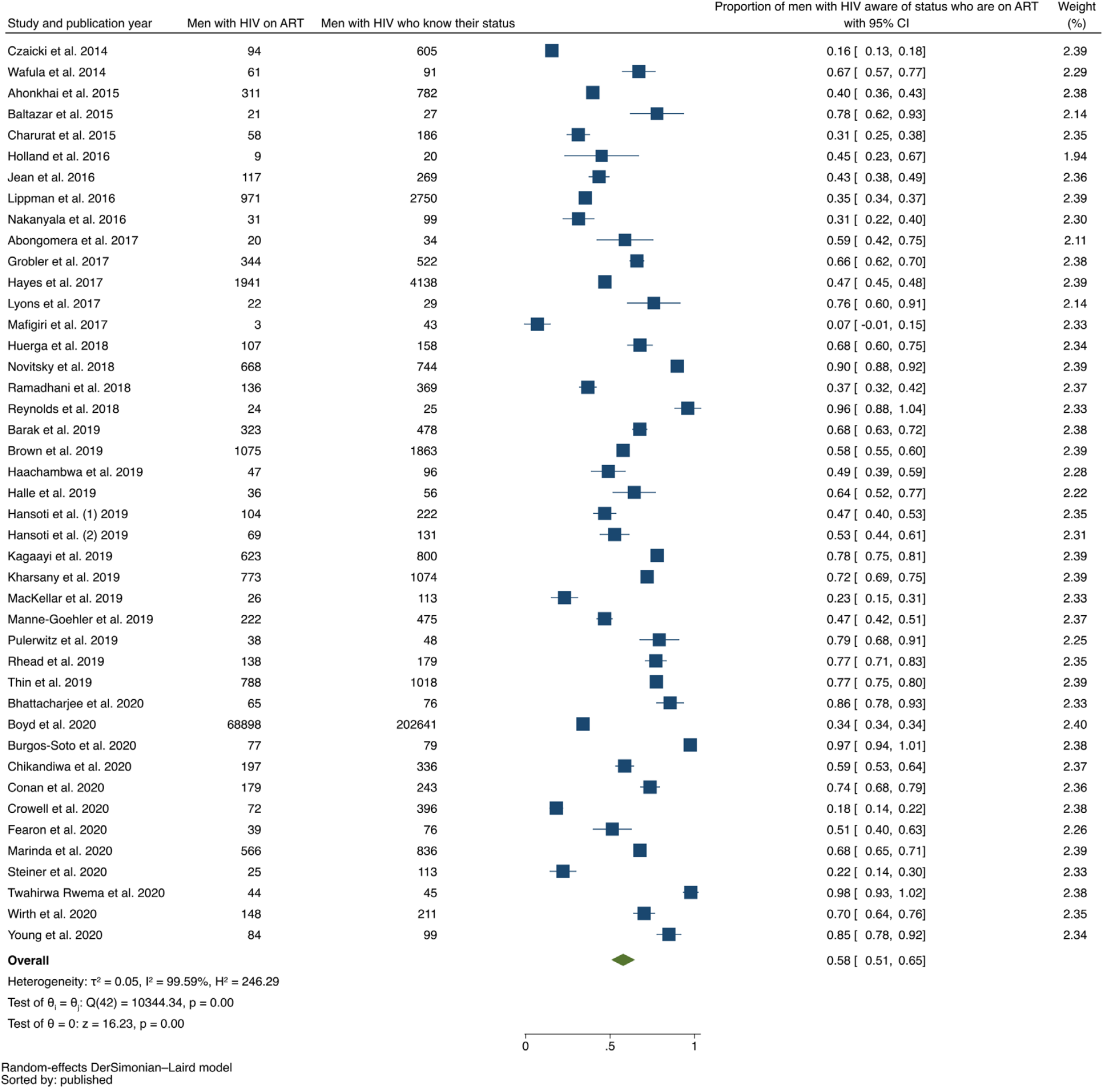
Forest plot of studies reporting data on proportion of men with HIV on ART out of all men with HIV who know their status, listed in ascending order of year of publication.

**Figure 4:**
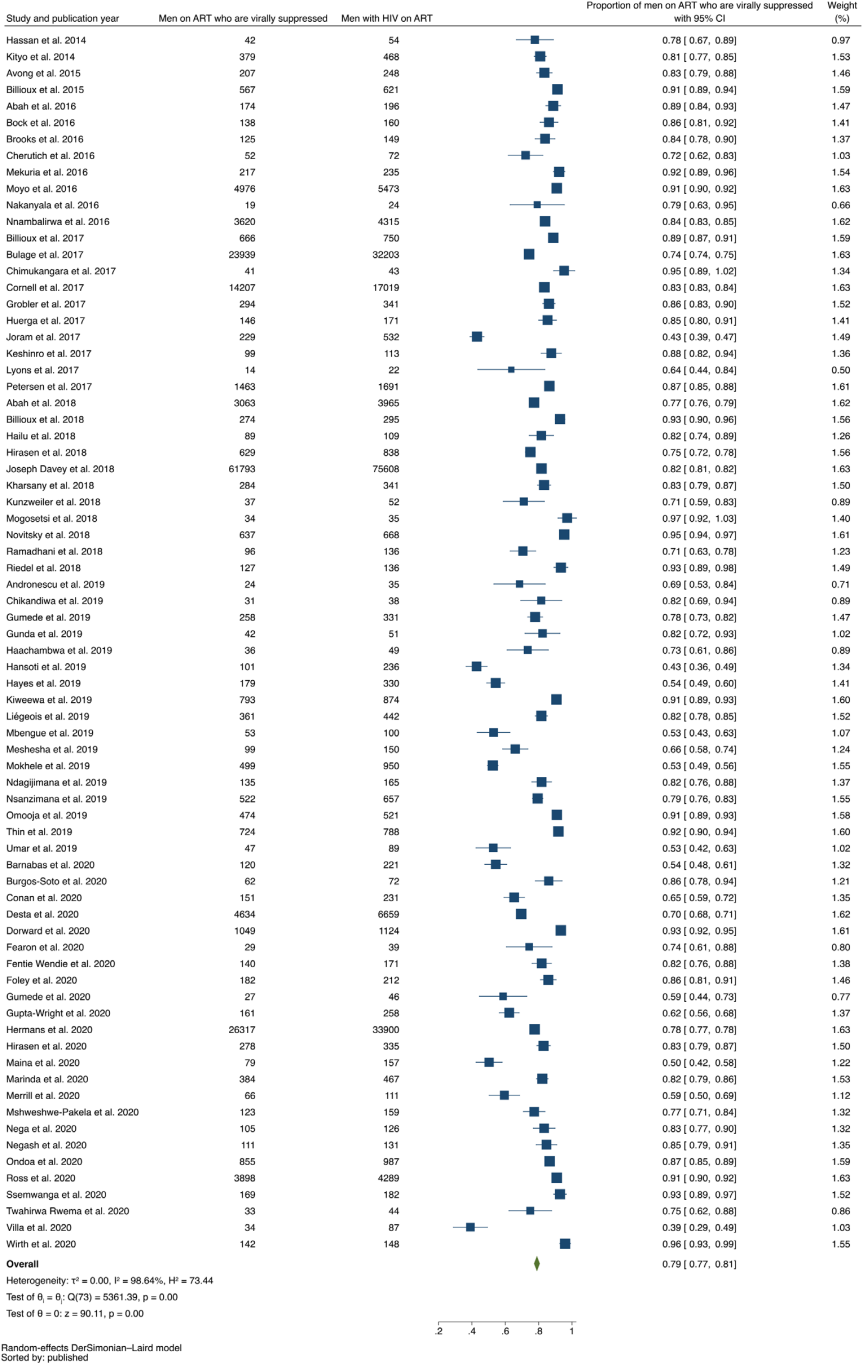
Forest plot of studies reporting data on proportion of men on ART who were virally suppressed out of all men with HIV on ART, listed in ascending order of year of publication.

In our analysis of data from 2009 (or prior) through 2020, the pooled prevalence of men with HIV who knew their positive status was 0.49 (95% confidence interval [CI], 0.41–0.58; range, 0.09–0.97) with evidence of high between‐study heterogeneity (*I* ^2^ = 99.68%) (Figure 2). The pooled prevalence of men with HIV on ART was 0.58 (95% CI, 0.51–0.65; range, 0.07–0.97), with evidence of high between‐study heterogeneity (*I* ^2^ = 99.59%) (Figure 3). The pooled prevalence of men with HIV on ART who achieved viral suppression was 0.79 (95% CI, 0.77–0.81; range, 0.39–0.97), with evidence of high between‐study heterogeneity (*I* ^2^ = 98.64%) (Figure 4).

In studies that enrolled both men and women with HIV, the proportions of men at each stage of the continuum were lower than those for women. A lower pooled proportion of men knew their HIV‐positive serostatus (0.53 [95% CI, 0.44–0.63; range, 0.09–0.97] among men vs. 0.66 [95% CI, 0.59–0.73; range, 0.13–0.98] among women; *p* = 0.04). A lower pooled proportion of men were on ART (0.54 [95% CI, 0.47–0.62; range, 0.07–0.97] among men vs. 0.62 [95% CI, 0.57–0.67; range, 0.17–0.99] among women; *p* = 0.09). A lower pooled proportion of men were virally suppressed (0.79 [95% CI, 0.77–0.81; range, 0.39–0.97] among men vs. 0.82 [95% CI, 0.80–0.83; range, 0.44–0.97] among women; *p* = 0.01) (Appendix).

Population, study setting and outcome measurement varied between studies (Appendix). The pooled proportion of men with HIV who knew their serostatus was lower in studies that focused exclusively on MSM compared with studies that did not exclusively focus on MSM (0.36 [95% CI, 0.23–0.49; range, 0.09–0.97; *I* ^2^ = 97.55] among MSM vs. 0.53 [95% CI, 0.44–0.62; range, 0.13–0.64; *I* ^2^ = 99.73] in mixed samples, *p* = 0.04). Similarly, the pooled proportion of men on ART who achieved viral suppression was lower in studies that focused exclusively on MSM compared with studies that did not (0.71 [95% CI, 0.66–0.77; range, 0.39–0.97; *I* ^2^<0.001] among MSM vs. 0.79 [95% CI, 0.78–0.81; range, 0.64–0.75; *I* ^2^ = 98.73] in mixed samples, *p*<0.001).

Other differences were noted (Appendix). Comparing data by time period, the pooled proportion of men on ART who were virally suppressed was higher in studies in which the median year of data collection was 2009–2016 versus in studies in which the median year of data collection was 2017–2020 (0.81 [95% CI, 0.79–0.83; range, 0.43–1.03; *I* ^2^ = 98.58] for 2009–2016 vs. 0.74 [95% CI, 0.68–0.79; range, 0.39–0.93; *I* ^2^ = 98.71] for 2017–2020, *p* = 0.02). The pooled proportion of men with HIV who knew their status was lower among studies in which knowledge was measured by self‐report prior to study testing versus studies in which knowledge was measured by other methods (0.46 [95% CI, 0.39–0.52; range, 0.09–0.86; *I* ^2^ = 99.07] based on self‐report prior to testing vs. 0.51 [95% CI, 0.42–0.60; range, 0.44–0.97; *I* ^2^ = 99.47] based on other methods, *p*<0.001). Finally, the pooled proportion of men with HIV on ART was lower in studies in which ART status was ascertained in a healthcare facility versus studies in which ART status was ascertained in a community setting (0.43 [95% CI, 0.34–0.51; range, 0.07–0.76; *I* ^2^ = 98.99] in healthcare facility‐based samples vs. 0.67 [95% CI, 0.58–0.75; range, 0.23–0.97; *I* ^2^ = 99.20] in community samples, *p*<0.001). We found no significant differences comparing by the other variables extracted, including study period, publication year and study quality.

The proportions of men at each stage of the continuum were generally lower in western and central Africa, although the only significant difference was found in comparing the pooled proportions of men on ART, which was higher in eastern and southern Africa in comparison to western and central Africa (0.60 [95% CI, 0.52–0.68; range, 0.07–0.98; *I* ^2^ = 99.66] in eastern and southern Africa vs. 0.47 [95% CI, 0.38–0.56; range, 0.31–0.76; *I* ^2^ = 88.44] in western and central Africa, *p* = 0.02). These comparisons likely were limited by the far fewer number of studies from western and central Africa.

### 3.2 Meta‐synthesis

The meta‐synthesis included 40 studies, representing views from 2683 participants in 10 countries (Table 2). The median number of participants was 38 (IQR, 25–93; range, 15–230). Participants ranged in age from 15 to 80 years.

**Table 2:** Qualitative studies of men's engagement in HIV care in sub‐Saharan Africa meeting inclusion criteria for meta‐synthesis (*N* = 40)

	Population	Country	Dates	Sample size	Male sample size	Component of HIV care continuum	Quality criteria
Adams et al. (2017)	Men with and without HIV and men with unknown status	Swaziland	2013–2014	76	76	Testing and treatment linkage, including Test and Start	All criteria met
Adeabgo et al. (2019)	People ages 18–79	South Africa	2017–2018	32	32	Testing and treatment linkage	Criteria partially met
Brown et al. (2019)	Men with HIV	Kenya and Uganda	2015–2016	190	190	Retention in care, including intervention strategies	All criteria met
Camlin et al. (2016)	Men with and without HIV and men with unknown status	Kenya and Uganda	2014	111	111	Testing, including barriers and intervention strategies	All criteria met
Chikovore et al. (2016)	Men with and without HIV and men with unknown status ages 17–70	South Africa	2013	20	10	Treatment as prevention	All criteria met
Conserve et al. (2019)	Men with unknown status ages 20–51	Tanzania	2015	146	23	Testing, including barriers and intervention strategies	All criteria met
Daniels et al. (2019)	Men who have sex with men (MSM) with HIV	South Africa	2013, 2017 and 2018	20	16	Antiretroviral therapy (ART) adherence	Criteria partially met
DiCarlo et al. (2014)	Men with known and unknown HIV status ages 24–57	Lesotho	2011	230	30	Testing, including barriers and intervention strategies	Criteria partially met
Fleming et al. (2016)	People who participated in gender and health equality‐based intervention ages 17–75	South Africa	2010	60	60	Overall HIV care engagement, including testing	All criteria met
Graham et al. (2018)	MSM with HIV ages 19–51	Kenya	2013–2014	30	30	Overall care engagement, including ART adherence	Criteria partially met
Hendrickson et al. (2019)	People with unknown status, with various levels of treatment engagement ages 25–49	Côte d'Ivoire	2016	227	227	ART use	Criteria partially met
Hill et al. (2018)	People with and without HIV ages 18–49	South Africa	2012–2014	25	25	Testing and treatment	All criteria met
Jennings et al. (2017)	Men who socialize at “camps” ages 20–51	Tanzania	2015	23	23	Self‐testing	All criteria met
Krakowiak et al. (2020)	Heterosexual couples with a median age of 28 years for men	Kenya	2015	42	21	Home‐based couple testing	All criteria met
Lavender et al. (2019)	Pregnant or postpartum women and male partners ages 20–48	Malawi and Kenya	2016–2017	76	36	Testing for antenatal partner	All criteria met
Mak et al. (2016)	Household community members age 15–49	Swaziland	2011–2012	33	33	Utilization of HIV services, including testing	All criteria met
Mantell et al. (2019)	Men who are actively participating in clinic‐based community ART refill groups age 18+	Zimbabwe	2017	147	118	ART refill groups	Criteria partially met
Martinez Perez et al. (2016)	People who denied HIV counselling and testing, couples who received counselling and testing, and HIV‐caregivers age 20–41	South Africa	2014–2015	20	9	Home self‐testing	All criteria met
Mburu et al. (2014)	People with HIV, their household members and healthcare providers age 30–64	Uganda	2010	65	40	Overall HIV care engagement and stigma	All criteria met
Micheni et al. (2017)	MSM age 18+ with a mean of 39	Kenya	2013–2014	29	14	ART adherence	All criteria met
Mooney et al. (2017)	Men with and without HIV with various levels of care engagement age 18–49	South Africa	2015	25	25	Treatment as prevention	All criteria met
Naugle et al. (2019)	Men with HIV and men with unknown HIV status age 25–49	Côte d'Ivoire	2016	227	227	Testing and treatment	Criteria partially met
Ndyabakira et al. (2019)	Men living in rural areas age 18–45+	Uganda	2016	60	60	Testing	All criteria met
Ogunbajo et al. (2018)	MSM with HIV age 18+	Ghana	2015	30	30	Overall HIV care engagement	All criteria met
Okal et al. (2020)	Men with HIV and health counsellors age 20–54	Kenya	2018	38	30	Testing	Criteria partially met
Orr et al. (2017)	Men age 18–44	South Africa	..	97	97	Testing and treatment initiation	Criteria partially met
Osingada et al. (2019)	Male football fans age 19–71	Uganda	2018	50	50	Testing	Criteria partially met
Rankin‐Williams et al. (2017)	Married subsistence farmers ages 23–50	Malawi	2014–2015	50	50	Testing	All criteria met
Rosen et al. (2020)	Fisherman with HIV ages 29–46	Uganda	2017–2018	25	15	ART sharing	All criteria met
Russell et al. (2019)	Low‐income people ages 30–74, some with HIV	Uganda	2011–2012	38	18	Treatment adherence	All criteria met
Sandfort et al. (2015)	MSM age 20–39, some with HIV	South Africa	2014	81	81	Testing	All criteria met
Schatz et al. (2018)	People with HIV ages 50–80	South Africa	2016–2017	21	11	Testing	All criteria met
Sileo et al. (Qualitative…) (2019a)	Fisherfolk with HIV on ART ages 20–50	Uganda	2016–2017	30	30	Treatment adherence	All criteria met
Sileo et al. (Masculinity…) (2019b)	Fisherfolk with HIV on ART ages 20–50	Uganda	2016–2017	30	30	Overall HIV care engagement	All criteria met
Skovdal et al. (2019)	Family members of men who died from HIV	Kenya, Malawi, South Africa, Tanzania, Uganda and Zimbabwe	2015–2016	26	26	HIV treatment engagement	All criteria met
Tibbels et al. (2019)	Men with HIV and men with unknown status age 25–49	Cote d'Ivoire	2016	227	227	Overall HIV care engagement	All criteria met
Tsang et al. (2019)	Male sex workers and MSM ages 19–38	Zimbabwe	2016–2017	15	15	MSM testing	All criteria met
Van Heerden et al. (2015)	Men ages 18–37 with unknown HIV status	South Africa	2011–2012	20	10	Testing	All criteria met
Wamoyi et al. (2017)	Men with HIV with various levels of care engagement	South Africa	2015–2016	107	55	Overall HIV care engagement	Criteria partially met
Zissette et al. (2016)	Men ages 24–80 with HIV on ART	South Africa	2014	21	21	Overall HIV care engagement	All criteria met

Our detailed review of the qualitative manuscripts identified 24 second‐order constructs, 11 third‐order constructs and three third‐order labels. Each third‐order label encompassed barriers to men's care engagement, as well as supportive factors, which allowed some men to engage in care despite these barriers (Table A3).

### 3.3 Theme 1: Perceived social norms

Most studies described how men believed that engaging in HIV care threatened their sense of social norms. Men may feel uncomfortable in health facilities perceived to be feminine spaces or that are staffed by women because, as one South African man explained, “men are not comfortable discussing their issues with women” [119] (p. 7). Moreover, HIV testing was felt to be a woman's responsibility because “men perceive their partners to be the ones that brought infection in the family” [118] (p. 9). In addition, participants described that HIV threatened men's ideals of strength, sexuality, livelihood, social standing and a fun lifestyle. HIV was “the end of your fun, the end of your joy,” imposing limitations on men's sexual choices because women will “run away” from a man who has HIV [192] (p. 6). Therefore, it was better not to know one's status. Participants also shared how engaging in care would compete with men's ability to work – something that many participants in Cote d'Ivoire described as being what “defines a man,” giving “social freedom…social status…and respect” [192] (p. 7). Men worried that engaging in HIV care would take away from time socializing with other men and “men activities,” leading them to feel “left behind, weak and incapable of fully being a man” [119] (p. 7).

However, many studies identified how some men were able to draw on positive coping skills to facilitate engagement in HIV care while still prioritizing their social roles. For example, a man in Uganda reported that knowing his positive status motivated him to “fight for my life” and “save money” in order to provide for his children [132] (p. 1204). Drawing on social support from other men was another coping strategy used by some participants. One man who was frequently ill shared how “my friends would advise me that why don't you go to a health facility such that you can be checked” [136] (p. 781). Participants also coped by seeing themselves as courageous and strong in the face of an HIV diagnosis. One man described, “[I have] ARVs as treatment and therefore I have no reason to be afraid.” [132] (p. 1207). Emphasizing his strong appearance, a fisherman in Uganda said, “I tell the people around that I am HIV infected…I show off because I look good” [132] (p. 1203).

### 3.4 Theme 2: Health system challenges

Numerous structural and social challenges related to the health system were described as barriers to accessing care. Social challenges included the experience or anticipation of poor treatment from providers with stigma towards people with HIV. One man shared his experience that “when [hospital staff] discover it is HIV, they give you a weird look….the staff laughs” [137] (p. 5). MSM described experiencing or anticipating stigma regardless of what their serostatus might be; as one MSM participant in Kenya said, “If I went to a health facility the moment I meet you I can tell how homophobic you are” [120] (p. 100). Other disincentives to seeking care were that participants doubted their HIV test results (“sometimes the person who does the test can be wrong” [137] (p. 6) ) or believed that there is no effective treatment for HIV. Structural challenges included men's concern about lacking privacy due to clinics’ physical layouts and procedures, such as a bench reserved for patients with HIV [137] (p. 7). Participants were also disincentivized to seek care at under‐resourced clinics experiencing clinician shortages or medication or test kit stockouts. A man with HIV in Côte d'Ivoire described that “…when there's no medication…I am discouraged” [137] (p. 8).

On the other hand, men described how strategies to mitigate these challenges did help them to access care. Convenient access to health facilities helped accommodate men's work schedules, such as one man's suggestion for facilities that “operate 24 hours” [194] (p. 14). Self‐testing and home‐testing were identified as quick and confidential ways for men to avoid having to return for follow‐up visits if their testing returned negative. One man in Tanzania described how self‐testing allowed him to avoid stigma because “none sees me while I test” [122] (p. 5). In addition to strategies promoting initial care engagement, personal support from providers and personally experiencing the effectiveness of ART helped to facilitate ongoing engagement in care. A man with HIV in South Africa described, “I believe that this treatment is good because…I look healthy and my body has recovered compared to last year” [128] (p. 279). One Ghanaian MSM participant related how a nurse “called me often and even when I am unable to go to the clinic, she'd get my medication for me and then I'll go collect it at her house” [129] (p. 834).

### 3.5 Theme 3: Poverty

Men explained how “the illness finds us in poverty” [136] (p. 780), making it challenging to overcome economic challenges associated with transport costs and medical expenses. A man in Côte d'Ivoire shared that men may opt for traditional healers because they “if they go to the hospital, the costs will be exorbitant” [137] (p. 8). Participants described the opportunity costs of engaging in HIV care, because such activities compete with the substantial time and energy needed for seeking employment and food [136] (p. 780).

However, strategies that made care more affordable helped offset these economic challenges. Specifically, home‐testing and self‐testing allowed men to avoid travelling and waiting in line. Some men also perceived self‐testing kits to be less expensive because “in private hospitals, you must pay to be tested” [122] (p. 5).

## 4 DISCUSSION

In this systematic review of 168 studies conducted in a wide range of settings across sub‐Saharan Africa, we found that health and social welfare systems have failed to achieve the UNAIDS 95‐95‐95 goals for men. Our meta‐analysis, combining data from 2009 (or prior) to 2020, showed that in aggregate over this time period, men have been behind in testing and treatment. Studies including only MSM found lower proportions in their knowledge of status and viral suppression as compared to the proportions for these goals in studies including all men. In studies comparing men and women, we found that men have had lower knowledge of HIV status and rates on ART, and slightly lower rates of viral suppression.

Our finding of lower proportions of men earlier in the care continuum contrasts with 2020 UNAIDS estimates from eastern and southern Africa [5], despite the fact that most studies in our meta‐analysis are from this region. It is more consistent with UNAIDS estimates from western and central Africa, showing that men have fallen behind especially in knowledge of status. We observed a lower rate of being on ART in western and central Africa as compared to eastern and southern Africa, whereas 2020 UNAIDS estimates found these rates to be comparable. These discrepancies may be explained by the fact that our meta‐analysis includes data over an extended time period of time, in contrast to a yearly estimate. They also may be explained by the marked heterogeneity in our studies. Lastly, the lack of statistically significant differences by region for knowledge of status and viral suppression, as predicted by current UNAIDS estimates, may be due to our small number of studies from western and central Africa.

Our findings regarding MSM support research showing that health systems in sub‐Saharan Africa inadequately engage MSM in achieving the 95‐95‐95 goals [7, 201]. A recent meta‐analysis on HIV testing and treatment among MSM in sub‐Saharan Africa similarly found that only 19% MSM with HIV knew their status, 60% of those MSM were on ART and 76% of those on ART achieved viral suppression – lower rates compared with the general population of all men [7]. There is an urgent need to better reach MSM, particularly as MSM are estimated to have a three‐fold greater prevalence of HIV compared with heterosexual men in sub‐Saharan Africa [202]. Complementing these findings, our meta‐synthesis identified unique barriers to care engagement for MSM. Intersecting stigmas attached to HIV and sexual minority status [127], consistent with prior research [203, 204], remain major challenges. Despite efforts to better reach MSM [205], there is an ongoing need for structural interventions to address large‐scale social forces beyond health systems.

In addition, we found that study setting, facility, age, employment status and migration status vary significantly among studies. More research is needed to focus on certain sub‐populations of men to understand where resources may be best utilized. For example, studies have noted the difficulty of engaging men in communities with substantial mobility [163, 206, 207]. At the same time, our meta‐synthesis revealed important areas of overlap among factors influencing engagement in HIV care for all men, suggesting opportunities for scalable interventions. Testing at venues telecasting football games [195], incentive‐based testing [193], self‐ and home testing [15], and outreach at bars and churches [124, 195] may help address the need for more men with HIV to know their status by incentivizing testing and bringing it to where men are in the community. Men‐only ART refill groups [191], expanded clinic opening hours [115], and social and livelihood interventions [208, 209] may address common concerns about stigma and the inconveniences and costs of care, helping more men with HIV to be on treatment. Gender‐transformative initiatives may also have an important role in helping men to reframe limiting norms and improve their testing and treatment outcomes [119].

**Figure 5:**
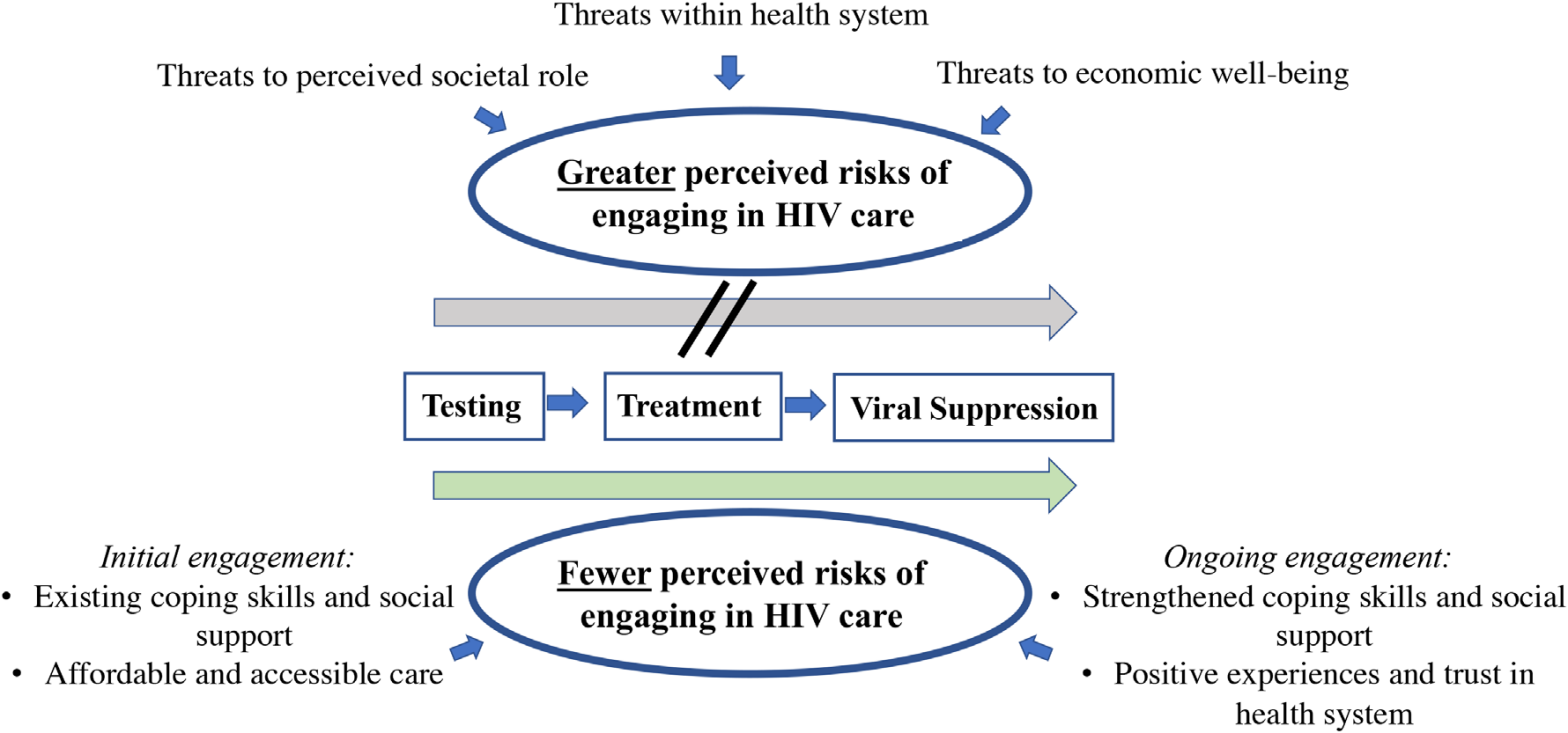
Conceptual model of men's engagement in the HIV care continuum.

Figure 5 depicts the conceptual model emerging from an integration of our quantitative and qualitative findings, drawing upon the framework of risk perception. For some men, the perceived risks of engaging in HIV care are substantial and influenced by perceived threats to their social role and economic wellbeing, as well as perceived threats within the health system. For other men – or for the same men at different points in time – these threats were mitigated by supportive factors, allowing the benefits of engaging in care to outweigh perceived risks. Supportive factors could facilitate initial care engagement (e.g. existing coping skills and social support, affordable and accessible care) as well as reinforce ongoing care engagement (e.g. strengthened coping skills and social support, positive experiences and trust in the health system). Coping is the process by which individuals manage their response to stressors [210, 211], and it encompasses both emotional coping strategies, such as feeling resilient, and problem‐focused coping strategies, such as turning to others for support [212, 213]. Positive coping strategies, both emotional and problem‐focused, have been shown to promote treatment decisions for persons with HIV [14, 214]. It is also possible that some individuals already in care may be encouraged to stay in care by experiencing or observing its benefits, such as a man who described recovering his physical health after being on treatment [128] (p. 279) or one who received invaluable treatment support from a nurse [129] (p. 834). Lastly, research has identified subgroups of patients with personal characteristics (e.g. younger men) that predispose them to progress more successfully through the care continuum [215, 216].

Our findings should be interpreted in light of some limitations. First, our meta‐analysis combined data over time from research studies rather than presenting annual data from programmatic surveillance. Therefore, our aggregate results are not directly comparable to annually updated surveillance data. Additionally, they may mask changes over this time period, in which there have been advances in HIV care. However, there may be advantages to our approach in that we included only research data rather than also estimates from modelling. It is also possible that our inclusion of some smaller studies may have captured important gaps throughout these regions that may be missed by larger population‐scale surveillance. Second, we found that the pooled estimates were characterized by a high degree of heterogeneity. However, this finding was not unexpected (and was consistent with heterogeneity estimates obtained in other recently published meta‐analyses), given that we had purposefully included a wide range of studies conducted in different settings. Third, we noted variation in measurement of the 95‐95‐95 goals, including in viral load thresholds. While the majority of studies used 1000 copies/ml as the threshold, the use of thresholds as high as 5000 copies/ml (or as low as 20 copies/ml) may over‐estimate (or under‐estimate) viral suppression in those studies. In addition, self‐report bias could have affected our pooled estimates of the first and second goals. Variation in outcome measurement will continue to hamper efforts to generate reliable estimates of men's engagement in the HIV care continuum and, therefore, efforts to develop scalable interventions to enhance HIV‐related outcomes in this population. We have found limited discussion of these issues in the literature [9, 217], and our finding highlights the need for harmonization of measurements across settings. Fourth, a majority of the screened studies did not meet our inclusion criteria. We found that the most common reasons for exclusion related to a lack of sex disaggregated data or outcomes that differed from the UNAIDS 95‐95‐95 goals, for example linkage to care but not being on ART. Despite our efforts to contact authors for additional data where possible, our results cannot account for data not included in the original manuscripts. Fifth, while we did not restrict inclusion to studies of cisgender men, there were very few studies that contained explicit specification. One included study explicitly included transgender men and one explicitly excluded transgender men. Thus, our review identifies this important gap in the literature. Lastly, we limited our search to PubMed and Embase for the meta‐synthesis given we anticipated that most qualitative studies would be in these biomedical and public health databases; however, it is possible that these searches missed relevant literature outside of these fields.

## 5 CONCLUSIONS

Men in sub‐Saharan Africa are behind in HIV testing and treatment, and MSM remain particularly vulnerable throughout the care continuum. Interventions that address men's perceived risks of care engagement by positively reframing living with HIV, providing social support, improving trust in and accessibility of the health system, and providing affordable care are needed to meet UNAIDS goals across sub‐Saharan Africa.

## COMPETING INTERESTS

ACT reports receiving a financial stipend from Elsevier, Inc. for his work as Co‐Editor in Chief of the journal *SSM‐Mental Health*. All other authors declare no competing interests.

## AUTHORS’ CONTRIBUTORS

MFN, ACT and ITK conceptualized this review and planned the analyses. MFN did the searches. MFN, OA and CP independently did all stages of screening and data extraction, and all data were checked by more than one author. MFN conducted all analyses with input from ACT and ITK. MFN interpreted the results and wrote the first draft of the manuscript, with contributions from OA and CP in creating the tables. ACT, ITK, BK, LL, SP and CGM made substantial intellectual contributions to the interpretation of the results and edited the manuscript. All authors read and approved the final version of the manuscript.

## FUNDING

This publication was made possible by the U.S. National Institutes of Health (NIH) T32AI007433 (MFN), K01MH119923 (LL) and R01MH113494 (ACT).

## DISCLAIMER

Its contents are solely the responsibility of the authors and do not necessarily represent the official views of the NIH. The funder had no role in study design, data collection, data analysis, data interpretation or writing of the report. The corresponding author had full access to all the data in the study and had final responsibility for the decision to submit for publication.

## Figures and Tables

**Figure A1:**
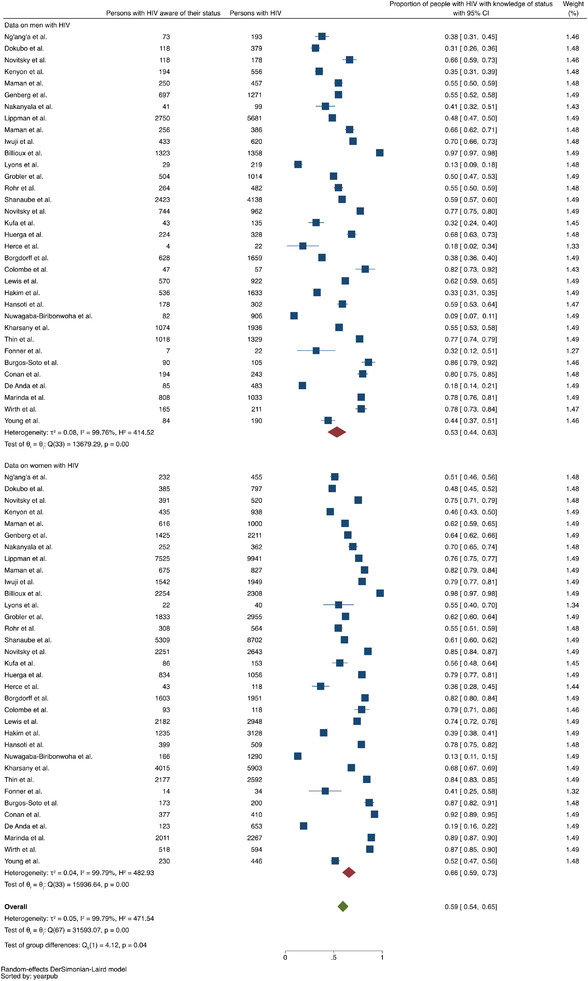
Forest plot comparing proportions of men and women with awareness of positive HIV status, including only studies reporting these data for both men and women (*N*=34 studies).

**Figure A2:**
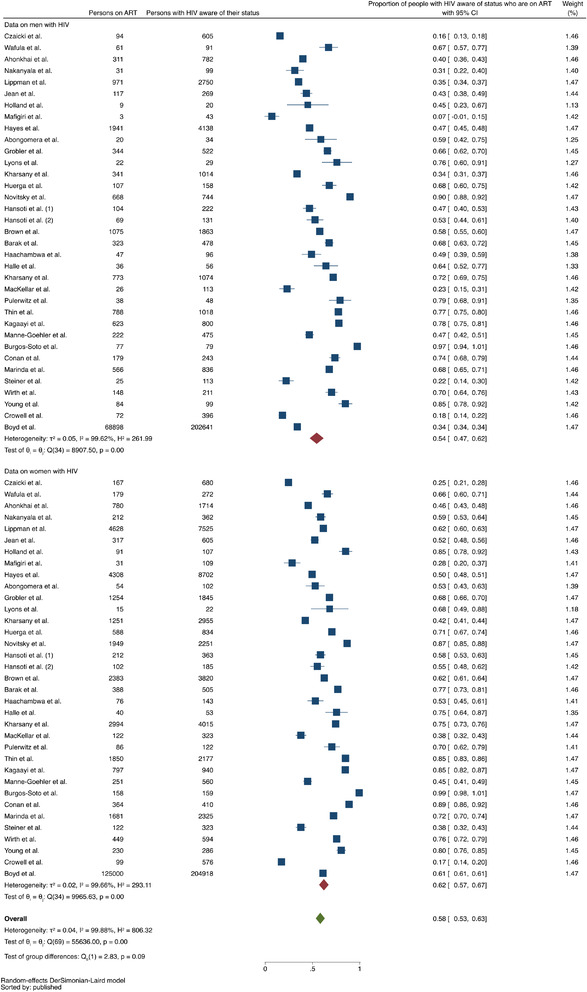
Forest plot comparing proportions of men and women on ART of those who are aware of their positive status, including only studies reporting these data for both men and women (*N*=35 studies).

**Figure A4:**
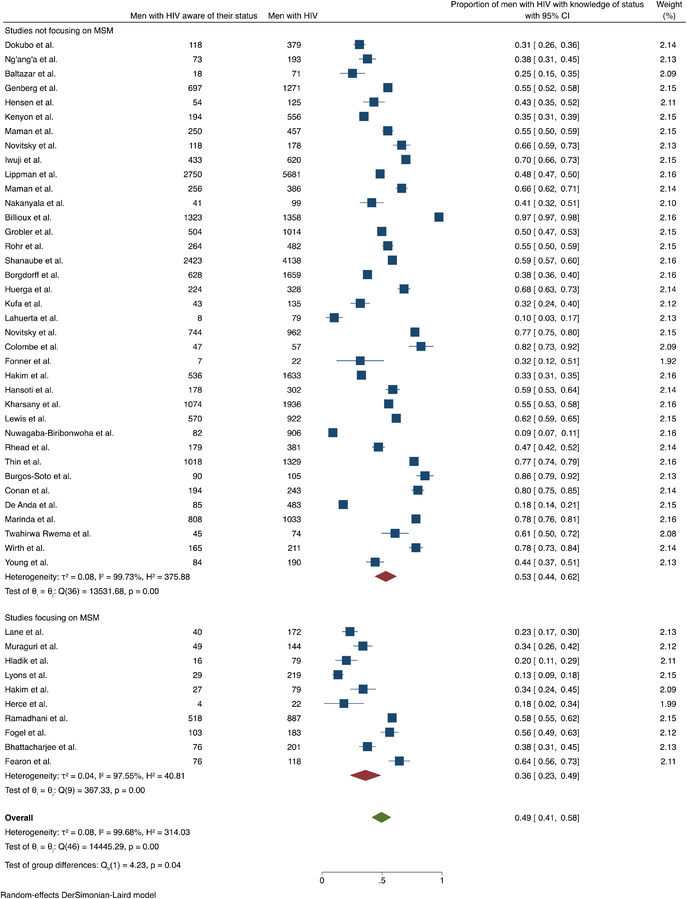
Forest plot comparing proportions of men with awareness of positive HIV status in studies focusing exclusively on MSM (*N*=10 studies) versus studies that did not focus exclusively on MSM (*N*=37 studies).

**Figure A5:**
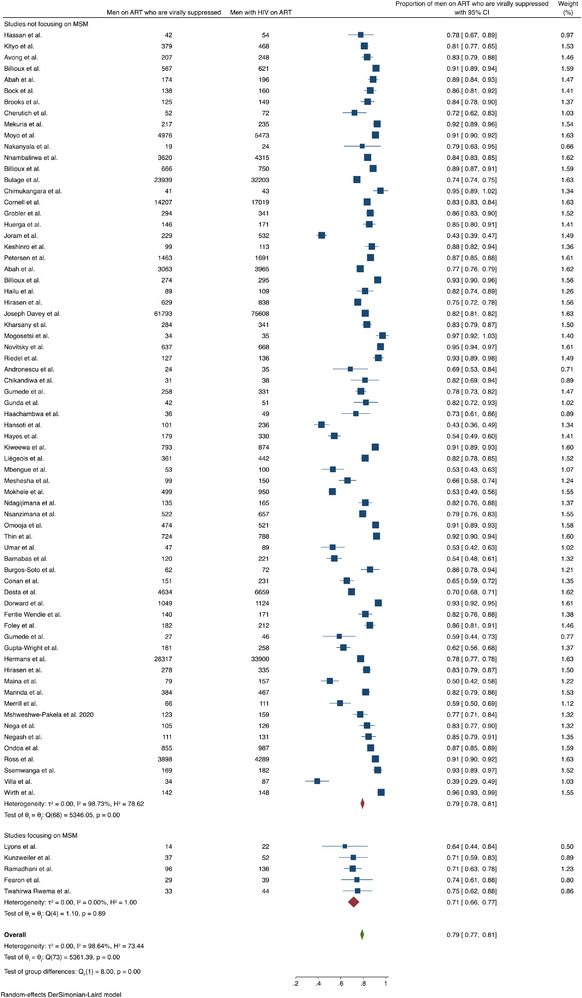
Forest plot comparing proportions of men on ART who are virally suppressed in studies focusing exclusively on MSM (*N*=5 studies) versus studies that did not focus exclusively on MSM (*N*=69 studies).

**Figure A6:**
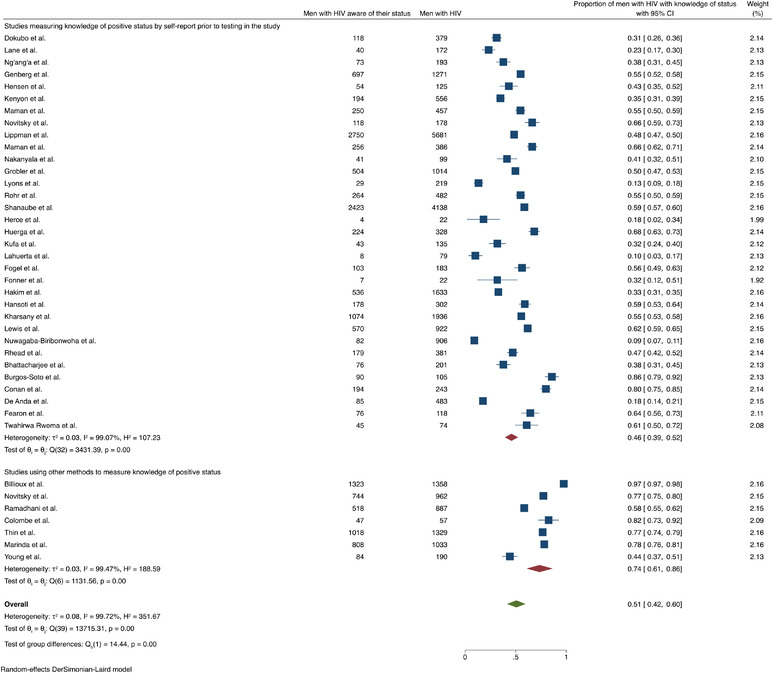
Forest plot comparing proportions of men with awareness of positive HIV status in studies in which knowledge of positive HIV status was measured by self‐report prior to testing (*N*=33 studies) versus studies in which knowledge was measured by other methods, such as awareness after testing in the study (*N*=7 studies).

**Figure A7:**
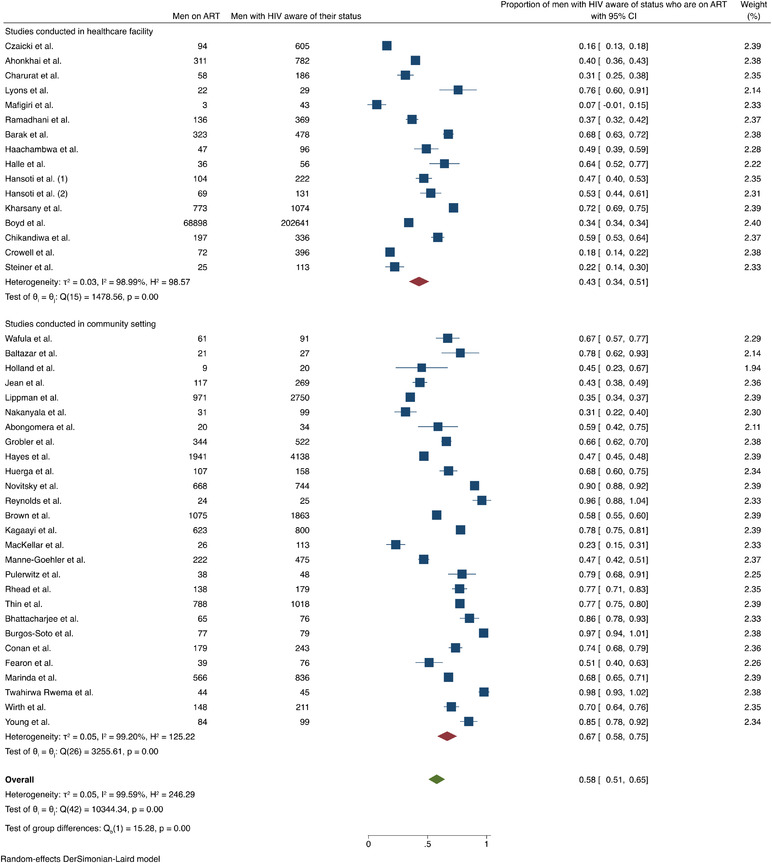
Forest plot comparing proportions of men with known HIV on ART in studies in which ART status was ascertained in a healthcare facility (*N*=16 studies) versus studies in which ART status was ascertained in a community setting (*N*=27 studies).

**Figure A8:**
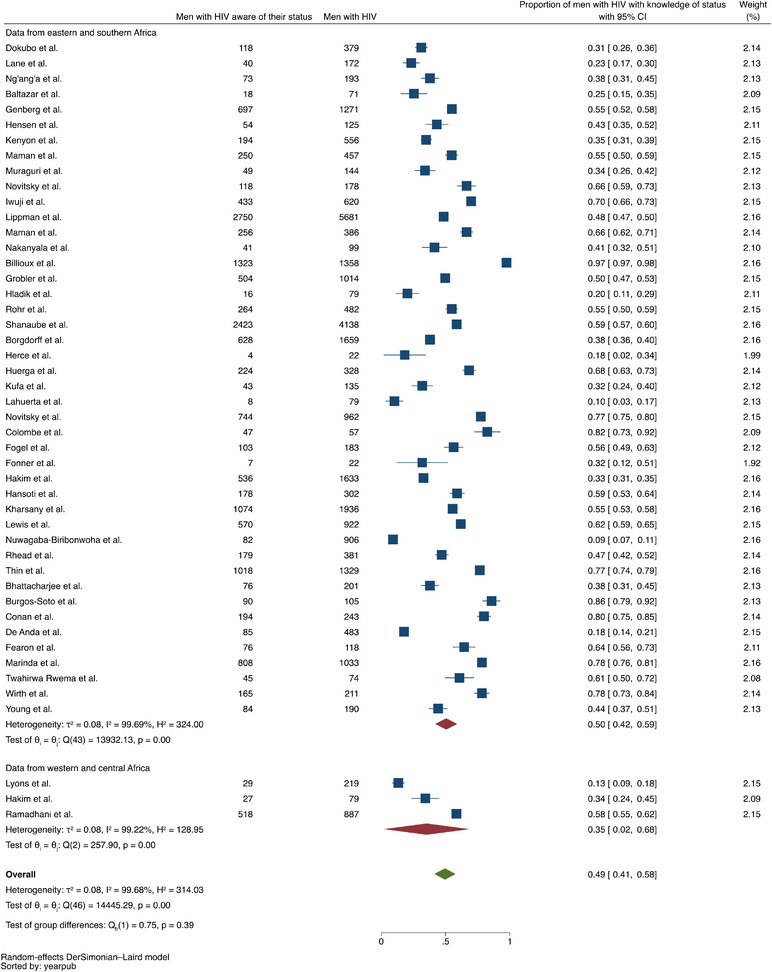
Forest plot comparing proportions of men with awareness of positive HIV status in studies conducted in eastern and southern Africa (*N*=44 studies) versus studies conducted in western and central Africa (*N*=3 studies).

**Figure A9:**
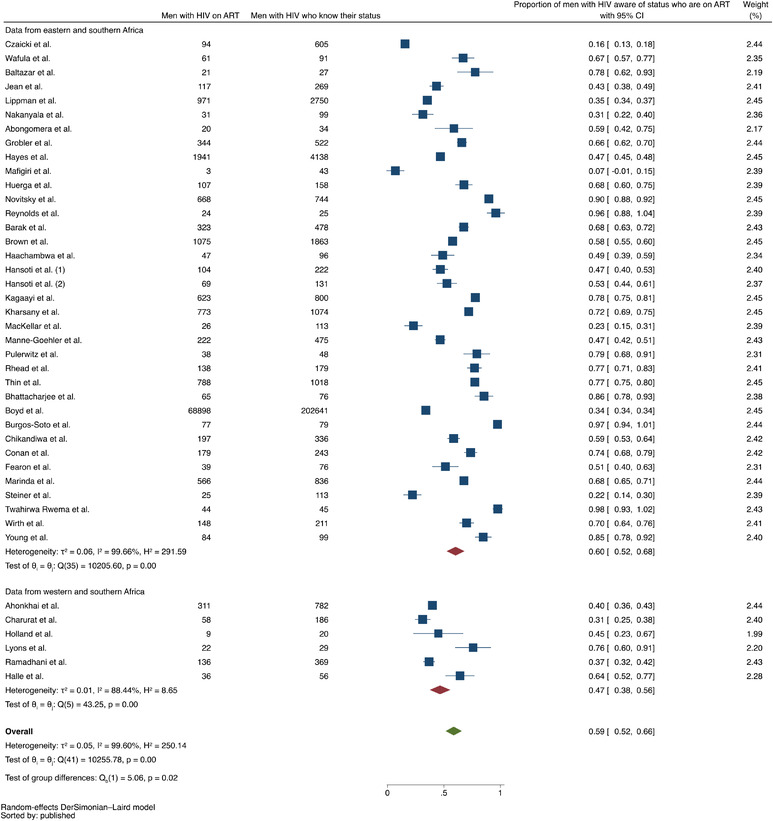
Forest plot comparing proportions of men with known HIV on ART in studies conducted in eastern and southern Africa (*N*=36 studies) versus studies conducted in western and central Africa (*N*=6 studies).

**Figure A10:**
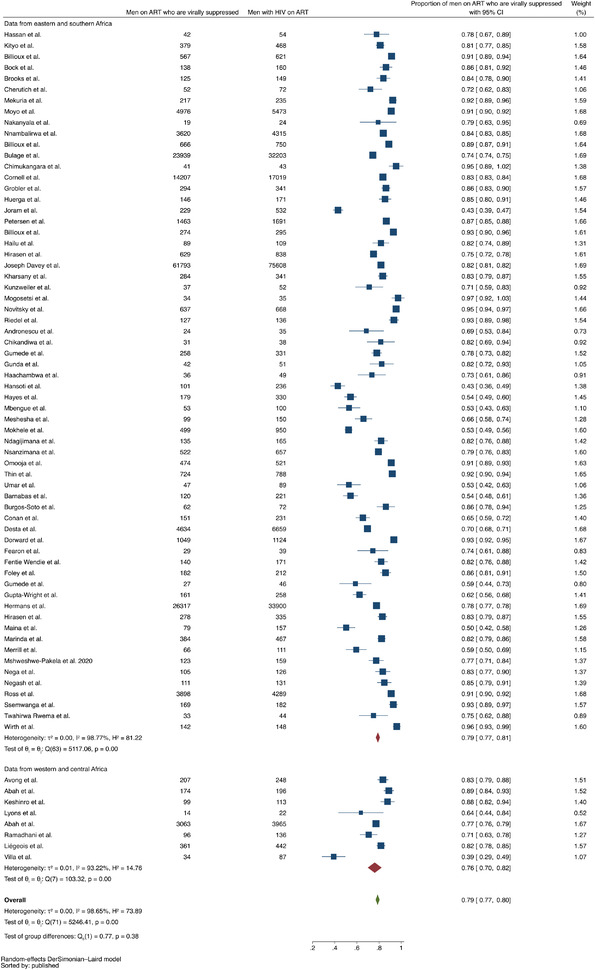
Forest plot comparing proportions of men on ART who are virally suppressed in studies conducted in eastern and southern Africa (*N*=64 studies) versus studies conducted in western and central Africa (*N*=8 studies).

**Figure A11:**
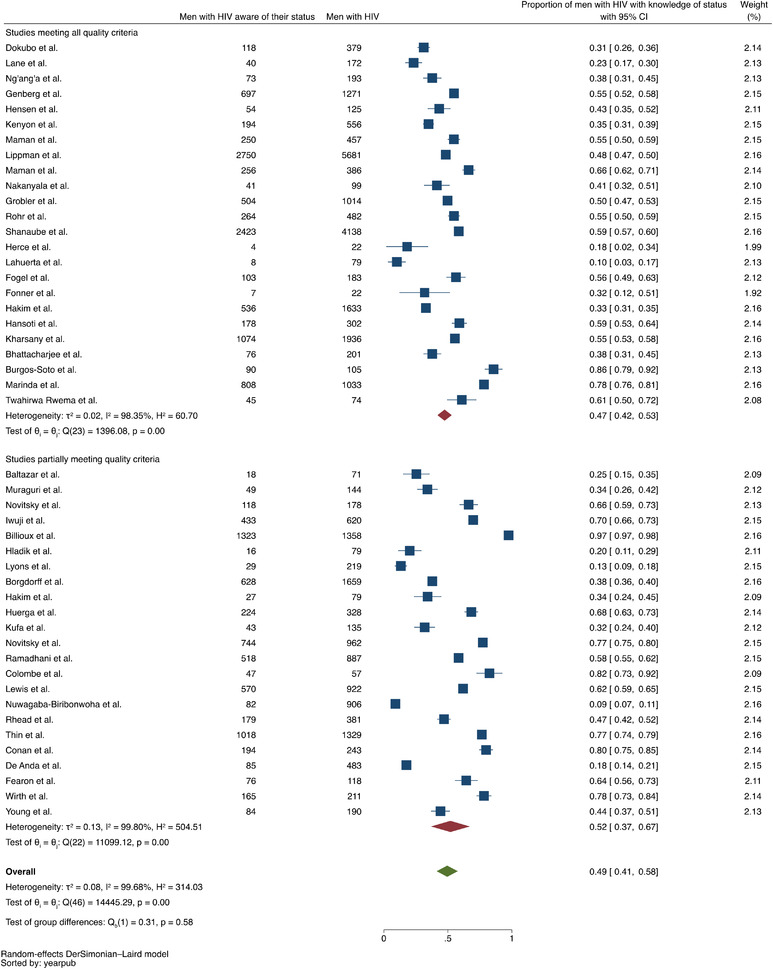
Forest plot comparing proportions of men with awareness of positive HIV status in studies in which all quality criteria are met (*N*=24 studies) versus studies in which quality criteria are partially met (*N*=23 studies).

**Figure A12:**
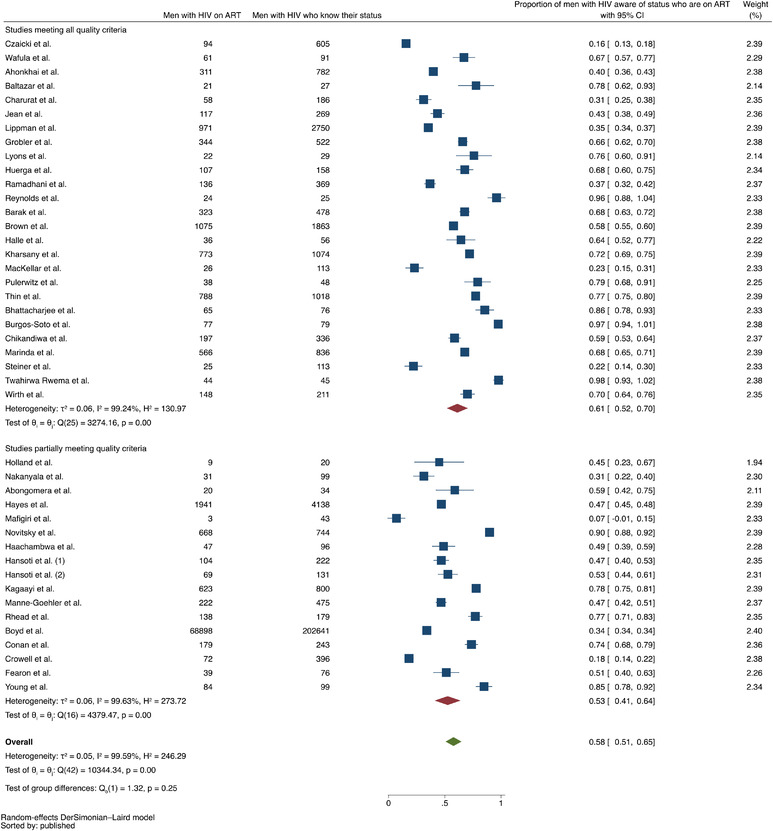
Forest plot comparing proportions of men with HIV on ART in studies in which all quality criteria are met (*N*=26 studies) versus studies in which quality criteria are partially met (*N*=17 studies).

**Figure A13:**
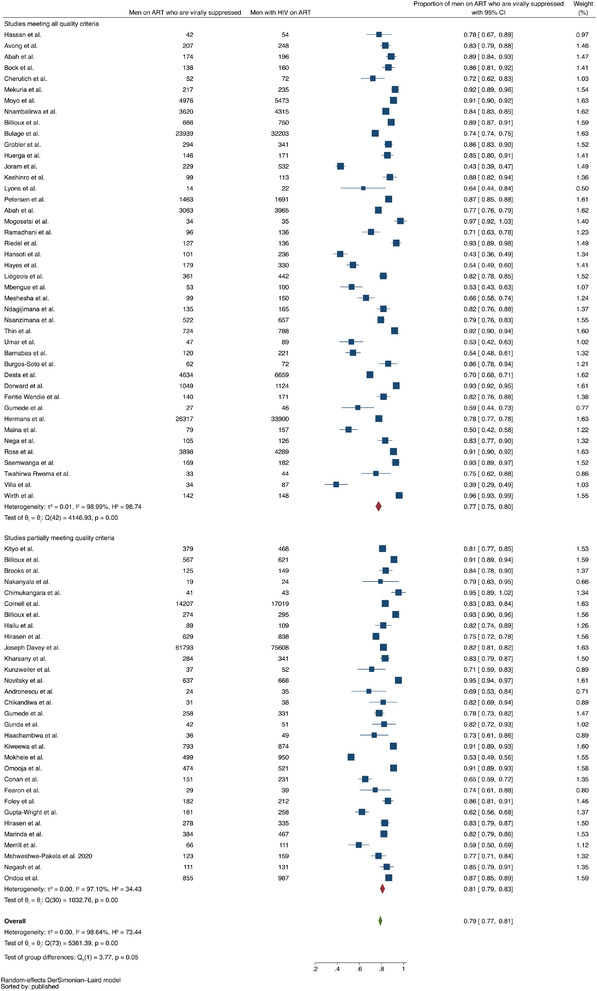
Forest plot comparing proportions of men on ART who are virally suppressed in studies in which all quality criteria are met (*N*=43 studies) versus studies in which quality criteria are partially met (*N*=31 studies).

**Figure A14:**
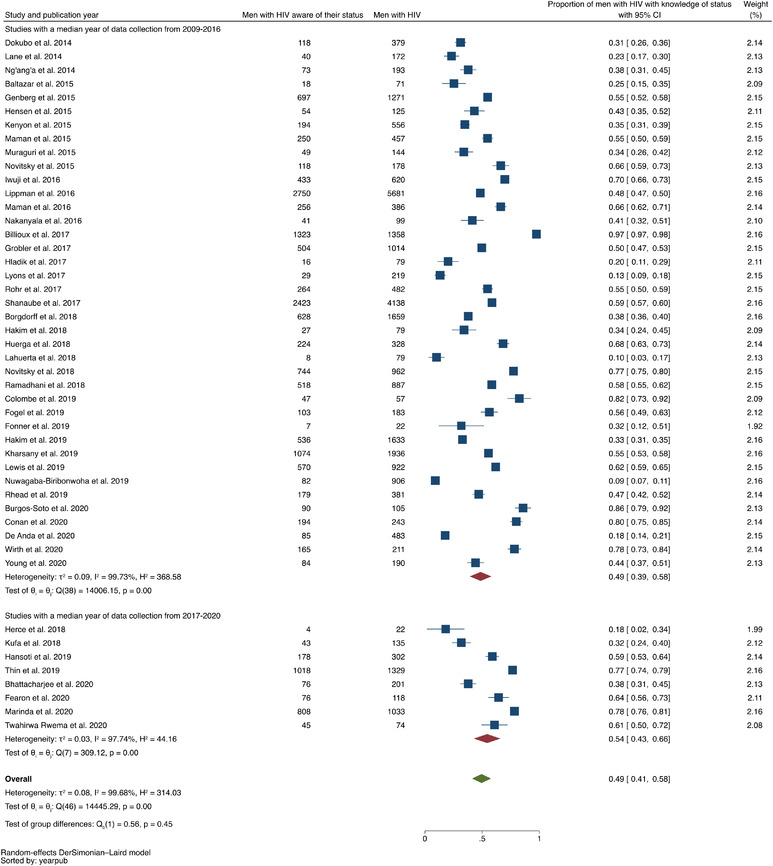
Forest plot comparing proportions of men with awareness of positive HIV status in studies in which the median year of data collection was between 2009 and 2016 (*N*=39 studies) versus in studies in which the median year of data collection was between 2017 and 2019 (*N*=8 studies).

**Figure A15:**
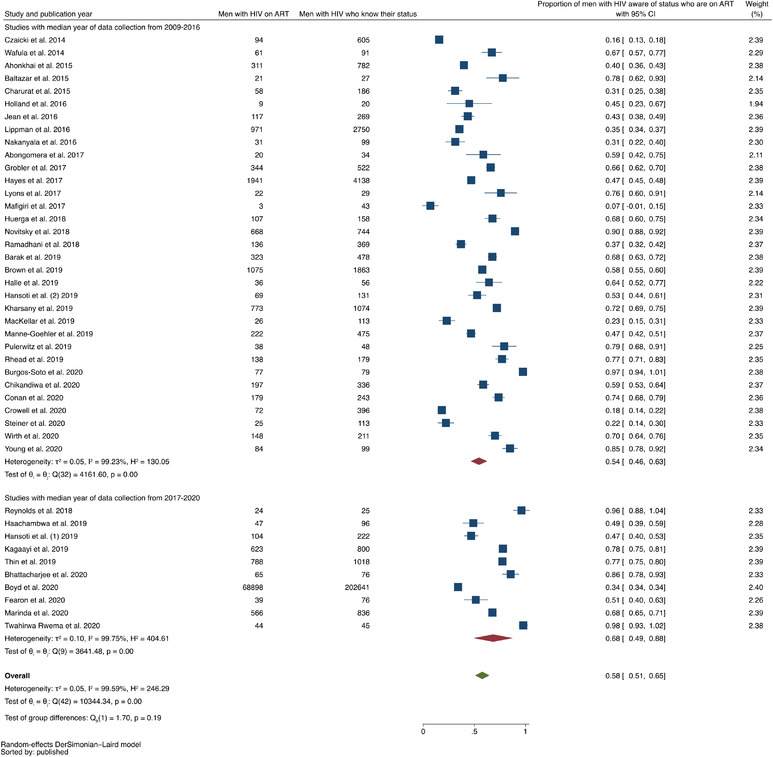
Forest plot comparing proportions of men with HIV on ART in studies in which the median year of data collection was between 2009 and 2016 (*N*=33 studies) versus studies in which the median year of data collection was between 2017 and 2020 (*N*=10 studies).

**Figure A16:**
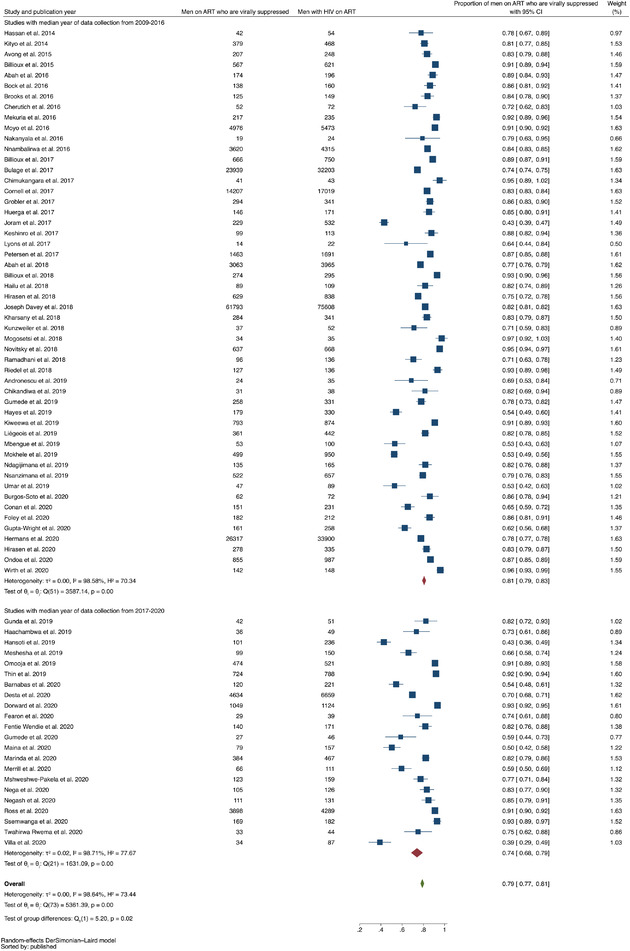
Forest plot comparing proportions of men on ART who are virally suppressed in studies in which the median year of data collection was between 2009 and 2016 (*N*=52 studies) versus studies in which the median year of data collection was between 2017 and 2020 (*N*=22 studies).

## Data Availability

All relevant data are within the manuscript and its Supporting Information files. No primary data were obtained for this study.

## Appendices A

### A.1 SEARCH TERMS USED IN PUBMED AND EMBASE

PubMed

(“HIV Infections”[Mesh] OR HIV[tiab])

AND

(“HIV testing”[tiab] OR “diagnosis” [MeSH Major Topic] OR “hiv status”[tiab] OR antiretroviral[tiab] OR HAART[tiab] OR ART[tiab] OR CART[tiab]) OR “HIV treatment” [tiab] OR “linkage to care”[tiab] OR “access to care”[tiab] OR “treatment access”[tiab]) OR adherence[tiab] OR “loss to follow up”[tiab] OR “viral suppression”[tiab] OR undetectable[tiab] OR “viral load”[tiab] OR “Viral Load”[Mesh] OR “treatment failure”[tiab] OR “virologic failure”[tiab] OR “90 90 90”[tiab] OR “care cascade”[tiab] OR “cascade of care”[tiab] OR “care continuum”[tiab] OR “continuum of care”[tiab] OR “fast track”[tiab])

AND

(“Africa South of the Sahara”[Mesh] OR africa[tiab] OR Cameroon[tiab] OR Central African Republic[tiab] OR Chad[tiab] OR Congo[tiab] OR Equatorial Guinea[tiab] OR Gabon[tiab] OR Burundi[tiab] OR Djibouti[tiab] OR Eritrea[tiab] OR Ethiopia[tiab] OR Kenya[tiab] OR Rwanda[tiab] OR Somalia[tiab] OR Sudan[tiab] OR Tanzania[tiab] OR Uganda[tiab] OR Angola[tiab] OR Botswana[tiab] OR Lesotho[tiab] OR Malawi[tiab] OR Mozambique[tiab] OR Namibia[tiab] OR South Africa[tiab] OR Swaziland[tiab] OR Zambia[tiab] OR Zimbabwe[tiab] OR Benin[tiab] OR Burkina Faso[tiab] OR Cape Verde[tiab] OR Cote d'Ivoire[tiab] OR Ivory Coast[tiab] OR Gambia[tiab] OR Ghana[tiab] OR Guinea[tiab] OR Guinea‐Bissau[tiab] OR Liberia[tiab] OR Mali[tiab] OR Mauritania[tiab] OR Niger[tiab] OR Nigeria[tiab] OR Senegal[tiab] OR Sierra Leone[tiab] OR Togo[tiab])

AND

(“2014/01/01”[PDAT] : “3000/12/31”[PDAT])

NOT

(Letter[pt] OR Editorial[pt] OR Review[pt] OR News[pt] OR Meta‐Analysis[pt] OR Guideline[pt])

EMBASE

(‘human immunodeficiency virus infection’/exp OR ‘human immunodeficiency virus infection’:ti,ab OR ‘human immunodeficiency virus infection’/mj) 

AND

(‘HIV testing’:ti,ab OR ‘diagnosis’/mj OR ‘hiv status’:ti,ab OR antiretroviral:ti,ab OR haart:ti,ab OR art:ti,ab OR cart:ti,ab OR ‘hiv treatment’:ti,ab OR ‘linkage to care’:ti,ab OR ‘access to care’:ti,ab OR ‘treatment access’:ti,ab OR ‘adherence’:ti,ab OR ‘loss to follow up’:ti,ab OR ‘viral suppression’:ti,ab OR ‘undetectable’:ti,ab OR ‘viral load’:ti,ab OR ‘viral load’/mj OR ‘treatment failure’:ti,ab OR ‘virologic failure’:ti,ab OR ‘90‐90‐90’:ti,ab OR ‘care cascade’:ti,ab OR ‘cascade of care’:ti,ab OR ‘care continuum’:ti,ab OR ‘continuum of care’:ti,ab OR ‘fast track’:ti,ab) 

AND

(‘africa’/exp OR ‘africa’:ti,ab) 

NOT

(‘letter’:it,pt OR ‘editorial’:it,pt OR ‘review’:it,pt OR ‘meta analysis’:it,pt OR ‘news’:it,pt OR ‘guideline’:it,pt) 

AND

[embase]/lim NOT ([embase]/lim AND [medline]/lim) 

AND

(2014:py OR 2015:py OR 2016:py OR 2017:py OR 2018:py OR 2019:py) 

### A.2 DEFINITIONS OF THE UNAIDS 95‐95‐95 GOALS

**Table A1:** Definition of numerator and denominator of each UNAIDS 95‐95‐95 goal

	Numerator	Denominator
First 95‐95‐95 goal	Persons with HIV aware of their serostatus	Persons with HIV
Second 95‐95‐95 goal	Persons with HIV on antiretroviral therapy	Persons with HIV aware of their serostatus
Third 95‐95‐95 goal	Persons with HIV on antiretroviral therapy and virally suppressed	Persons with HIV on antiretroviral therapy

### A.3 TABLE OF INCLUDED STUDIES IN META‐ANALYSIS

**Table A2:** Studies reporting on 95‐95‐95 goal(s) meeting inclusion criteria for meta‐analysis (*N*=129)

	Study population	Study type	Dates	Country	Total number of study participants	95‐95‐95 Goal reported	Proportion of men meeting 95‐95‐95 goal(s)	Quality criteria
Abah et al. (2016)	Individuals living with HIV initiated on NNRTI‐based ART, median age 34 years (interquartile range [IQR] 29–41 years)	Retrospective cohort study	2009–2010	Nigeria	588	Third	0.89	All criteria met
Abah et al. (2019)	Individuals living with HIV on first‐line ART, median age 34 years (IQR 29–41 years)	Retrospective cohort study	2004–2012	Nigeria	12,115	Third	0.77	All criteria met
Abongomera et al. (2017)	Individuals reporting HIV testing	Cross‐sectional study	2015	Uganda	2124	Second	0.59	Criteria partially met
Ahonkhai et al. (2015)	Individuals living with HIV on ART, median age 32 years (IQR 27–39 years)	Retrospective cohort study	2009–2011	Nigeria	2496	Second	0.40	All criteria met
Andronescu et al. (2019)	Individuals living with HIV initiating third‐line ART, median age 40 years (IQR 18–49 years)	Retrospective cohort study	2012–2015	Zambia	80	Third	0.69	Criteria partially met
Avong et al. (2015)	Individuals living with HIV on ART, age 21–60 years	Cross‐sectional study	2004–2010	Nigeria	502	Third	0.83	All criteria met
Baltazar et al. (2015)	Men working in mines, age 23–68 years	Cross‐sectional study	2012	South Africa and Mozambique	432	First and second	0.25 and 0.78	Criteria partially met
Barak et al. (2019)	Individuals with median age 51 years (IQR 34–71 years)	Prospective review cohort study	2015–2017	Botswana	1969	Second	0.68	All criteria met
Barnabas et al. (2020)	Individuals living with HIV, age ≥18 years	Household‐randomized unblinded trial	2016–2019	South Africa and Uganda	1315	Third	0.54	All criteria met
Bhattacharjee et al. (2020)	Men who have sex with men (MSM), age ≥15 years	Cross‐sectional bio‐behavioural survey	2019	Kenya	1200	First and second	0.38 and 0.86	All criteria met
Billioux et al. (2015)	Individuals living with HIV initiated on first‐line ART, median age 33 years (IQR 28–40 years)	Prospective cohort study	2005–2011	Uganda	1841	Third	0.91	Criteria partially met
Billioux et al. (2017)	Individuals living with HIV enrolled in care, age 15–49 years	Retrospective longitudinal cohort study	2013–2015	Uganda	3666	First and third	0.97 and 0.89	Criteria partially met
Billioux et al. (2018)	Individuals living with HIV enrolled in care, age 15–49 years	Census surveillance cohort study	2015–2016	Uganda	1554	Third	0.93	Criteria partially met
Bock et al. (2016)	Individuals living with HIV, age 18–49 years	Cross‐sectional study	2012	Swaziland	927	Third	0.86	All criteria met
Borgdorff et al. (2018)	Individuals aged 15–64 years	Population‐based cross‐sectional survey study	2011–2012 and 2016	Kenya	28,486	First	0.38	Criteria partially met
Boyd et al. (2020)	Individuals living with HIV, age ≥15 years	Cross‐sectional report	2018–2019	Zambia	248,002	Second	0.34	Criteria partially met
Brooks et al. (2016)	Individuals living with HIV initiated on first‐line ART, median age 41 years (IQR 23–82 years)	Cross‐sectional study	2012–2013	Kenya	333	Third	0.84	Criteria partially met
Brown et al. (2019)	Individuals living with HIV enrolled in care, age ≥15 years	Prospective cohort study	2014–2015	Kenya and Uganda	5683	Second	0.58	All criteria met
Bulage et al. (2017)	Individuals living with HIV on ART, mostly aged 35+ years	Cross‐sectional study	2014–2015	Uganda	100,678	Third	0.74	All criteria met
Burgos‐Soto et al. (2020)	Individuals aged 15–69 years	Household‐based cross‐sectional survey study	2016	Uganda	1738	First, second and third	0.86, 0.97 and 0.86	All criteria met
Charurat et al. (2015)	MSM living with HIV, age ≥16 years	Prospective cohort study	2013–2014	Nigeria	186	Second	0.31	All criteria met
Cherutich et al. (2016)	Individuals living with HIV, age 15–64 years	Cross‐sectional study	2012–2013	Kenya	617	Third	0.72	All criteria met
Chikandiwa et al. (2019)	Men living with HIV, age ≥18 years	Prospective cohort study	2011–2012	South Africa	304	Third	0.82	Criteria partially met
Chikandiwa et al. (2020)	Men living with HIV, age ≥18 years	Prospective cohort study	2012–2013	South Africa	304	Second	0.59	All criteria met
Chimukangara et al. (2017)	Individuals living with HIV on ART, median age 43 years (95% CI 39–44 years)	Cross‐sectional study	2014	Zimbabwe	143	Third	0.95	Criteria partially met
Colombe et al. (2020)	Individuals living with HIV, median age 36 years (IQR 27–46 years)	Community‐based prospective cohort study	2006–2016	Tanzania	175	First	0.82	Criteria partially met
Conan et al. (2020)	Individuals age ≥15 years old	Population‐based cross‐sectional survey study	2016	Zimbabwe	4979	First, second and third	0.80, 0.74 and 0.65	Criteria partially met
Cornell et al. (2017)	Individuals living with HIV	Prospective cohort study	2004–2015	South Africa	72,812	Third	0.83	Criteria partially met
Crowell et al. (2020)	Individuals living with HIV, median age 35.7 years (IQR 29.7–42.7) years	Retrospective cohort study	2013–2019	Uganda, Kenya, Tanzania and Nigeria	972	Second	0.18	Criteria partially met
Czaicki et al. (2014)	Individuals age ≥16 years	Prospective cohort study	2011–2012	Zambia	21,612	Second	0.16	All criteria met
De Anda et al. (2020)	Individuals living with HIV, age ≥18 years old	Cross‐sectional study	2015–2016	Kenya	1136	First	0.18	Criteria partially met
Desta et al. (2020)	Individuals living with HIV, age ≥15 years	Retrospective cross‐sectional study	2015–2019	Ethiopia	19,525	Third	0.7	All criteria met
Dokubo et al. (2014)	Individuals living with HIV, age 15–49 years	Cross‐sectional study	2009	Mozambique	1182	First	0.31	All criteria met
Dorward et al. (2020)	Individuals living with HIV on ART, age >15 years	Retrospective cohort study	2016–2019	South Africa	4952	Third	0.93	All criteria met
Fearon et al. (2020)	MSM and transgender individuals, age ≥18 years	Cross‐sectional study	2017	South Africa	301	First, second and third	0.64, 0.51 and 0.74	Criteria partially met
Fentie Wendie et al. (2020)	Individuals living with HIV on ART, age ≥15 years old	Retrospective cohort study	2018–2019	Ethiopia	384	Third	0.82	All criteria met
Fogel et al. (2019)	MSM and transgender women (TGW) who have sex with men, age 18–44 years	Prospective cohort study	2015–2016	Kenya, Malawi and South Africa	183	First	0.56	All criteria met
Foley et al. (2020)	Individuals living with HIV on ART, median age 32 years (IQR 26–40) years	Prospective cohort study	–	Uganda	657	Third	0.85	Criteria partially met
Fonner et al. (2019)	Individuals age ≥18 years old	Cross‐sectional study	2006	Tanzania	644	First	0.32	All criteria met
Genberg et al. (2015)	Individuals living with HIV enrolled in care, median age 36 years (IQR 30–45 years)	Retrospective cohort study	2004–2014	Kenya	3482	First	0.54	All criteria met
Grobler et al. (2017)	Individuals aged 15–49 years	Household‐based cross‐sectional survey study	2014–2015	South Africa	9812	First, second and third	0.50, 0.66 and 0.86	All criteria met
Gumede et al. (2019)	Individuals living with HIV on second‐line ART mostly 25+ years	Prospective cohort study	2014–2015	South Africa	825	Third	0.78	Criteria partially met
Gumede et al. (2020)	Individuals living with HIV on second‐line ART, median age 42 years (IQR 36–47 years)	Cross‐sectional study	2018	South Africa	149	Third	0.59	All criteria met
Gunda et al. (2019)	Individuals living with HIV on second‐line ART, median age 48 years (IQR 41–54 years)	Unmatched case–control study	2017–2018	Tanzania	197	Third	0.82	Criteria partially met
Gupta‐Wright et al. (2020)	Individuals living with HIV on ART, age ≥18 years	Observational cohort study	2015–2017	Malawi	1316	Third	0.62	Criteria partially met
Haachambwa et al. (2019)	Individuals living with HIV, age ≥18 years	Prospective cohort study	2017–2018	Zambia	239	Second and third	0.49 and 0.74	Criteria partially met
Hailu et al. (2018)	Individuals living with HIV on ART, age 10–63 years	Retrospective cohort study	2008–2016	Ethiopia	260	Third	0.82	Criteria partially met
Hakim et al. (2018)	MSM and TGW, age ≥18 years	Cross‐sectional study	2014–2015	Mali	552	First	0.34	Criteria partially met
Hakim et al. (2019)	Individuals age ≥13 years	Cross‐sectional study	2011–2013	Uganda	12,233	First	0.33	All criteria met
Halle et al. (2019)	Individuals living with HIV aged 22–82 years	Retrospective cohort study	2007–2013	Cameroon	156	Second	0.64	All criteria met
Hansoti et al. (2019)	Individuals mostly aged 20+ years	Cross‐sectional study	2017–2018	South Africa	2901	First, second and third	0.59, 0.58 and 0.43	Criteria partially met
Hansoti et al. (2019)	Individuals mostly aged 20+ years	Cross‐sectional serosurvey study	2016	South Africa	2100	Second	0.55	Criteria partially met
Hassan et al. (2014)	Individuals living with HIV on ART, median age 38.5 years (IQR 32.2–44.8 years)	Cross‐sectional study	2008–2011	Kenya	232	Third	0.78	All criteria met
Hayes et al. (2017)	Individuals age ≥18 years	Cluster randomized trial	2013–2015	Zambia	121,130	Second	0.47	Criteria partially met
Hayes et al. (2019)	Individuals aged 18–44 years	Community‐randomized trial	2013–2018	Zambia and South Africa	48,301	Third	0.54	All criteria met
Hensen et al. (2015)	Men aged 15–60 years	Cluster randomized stepped‐wedge trial	2011–2012	Zambia	2828	First	0.43	All criteria met
Herce et al. (2018)	MSM, TGW, and female sex workers (FSW), mostly >25 years	Cross‐sectional study	2016–2017	Malawi and Angola	1924	First	0.18	All criteria met
Hermans et al. (2020)	Individuals living with HIV on first‐line ART, median age 35.7 years (IQR 29.9–43.0 years)	Retrospective cohort study	2007–2018	South Africa	104,719	Third	0.78	All criteria met
Hirasen et al. (2018)	Individuals living with HIV initiated on first‐line ART, median age 37.8 years (IQR 31.7–45.0 years)	Retrospective cohort study	2011–2015	South Africa	3151	Third	0.75	Criteria partially met
Hirasen et al. (2020)	Individuals living with HIV on ART, age ≥18 years	Prospective cohort study	2014–2017	South Africa	2410	Third	0.83	Criteria partially met
Hladik et al. (2017)	MSM with median age 23 years (IQR 21–26 years)	Cross‐sectional study	2012–2013	Uganda	608	First	0.2	Criteria partially met
Holland et al. (2016)	MSM and FSW, age ≥18 years	Prospective cohort study	2013	Burkina Faso and Togo	2738	Second	0.45	Criteria partially met
Huerga et al. (2017)	Individuals aged 15–59 years	Cross‐sectional survey study	2013	South Africa	5649	Third	0.85	All criteria met
Huerga et al. (2018)	Individuals aged 15–59 years	Cross‐sectional study	2013	South Africa	5649	First and second	0.68 and 0.68	Criteria partially met
Iwuji et al. (2016)	Individuals aged ≥16 years	Cluster‐randomized trial	2012–2014	South Africa	9927	First	0.7	Criteria partially met
Jean et al. (2016)	Individuals aged 18–49 years	Cross‐sectional survey study	2012	South Africa	6766	Second	0.43	All criteria met
Joram et al. (2017)	Individuals living with HIV on ART, age 2–80 years	Retrospective cohort study	2013–2014	Kenya	1859	Third	0.43	All criteria met
Joseph Davey et al. (2018)	Individuals living with HIV initiated on first‐line ART, median age 37.8 years (IQR 31.7–45.0 years)	Retrospective cohort study	2004–2016	South Africa	244,370	Third	0.82	Criteria partially met
Kagaayi et al. (2019)	Individuals aged 15–49 years	Population‐based prospective cohort study	2016–2017	Uganda	8942	Second	0.78	Criteria partially met
Kenyon et al. (2015)	Individuals living with HIV, age 15–59 years	Cross‐sectional study	2011	Uganda	1495	First	0.35	All criteria met
Keshinro et al. (2017)	Individuals living with HIV on first‐line ART, age ≥18 years	Cross‐sectional study	2012	Nigeria	325	Third	0.88	All criteria met
Kharsany et al. (2018)	Individuals aged 15–49 years	Cross‐sectional survey study	2014–2015	South Africa	9812	Third	0.83	Criteria partially met
Kharsany et al. (2019)	Individuals aged 15–49 years	Community‐based longitudinal study	2014–2015	South Africa	20,048	First and second	0.55 and 0.72	All criteria met
Kityo et al. (2014)	Individuals living with HIV on ART, mostly aged 30+ years	Open randomized trial	2003–2009	Uganda and Zimbabwe	1896	Third	0.81	Criteria partially met
Kiweewa et al. (2019)	Individuals living with HIV, age ≥18 years	Prospective cohort study	2013–2017	Nigeria, Uganda, Kenya and Tanzania	2054	Third	0.91	Criteria partially met
Kufa et al. (2018)	Individuals, median age 26 years (IQR 23–32 years)	Cross‐sectional study	2017–2018	South Africa	1054	First	0.32	Criteria partially met
Kunzweiler et al. (2018)	MSM living with HIV, median age 27 years (IQR 22–32 years)	Prospective cohort study	2015–2016	Kenya	63	Third	0.71	Criteria partially met
Lahuerta et al. (2018)	MSM, age ≥18 years	Cross‐sectional study	2014–2015	Kenya, Mozambique, Rwanda and Tanzania	552	First	0.1	All criteria met
Lane et al. (2014)	MSM, age ≥18 years	Cross‐sectional study	2012–2013	South Africa	605	First	0.23	All criteria met
Lewis et al. (2019)	Individuals aged 15–49 years	Cross‐sectional study	2015–2016	South Africa	10,236	First	0.62	Criteria partially met
Liégeois et al. (2019)	Individuals living with HIV on ART, median age 41 years (IQR 35–49 years)	Cross‐sectional study	2014	Cameroon	1700	Third	0.82	All criteria met
Lippman et al. (2016)	Individuals aged 18–49 years	Population‐based household cross‐sectional survey study	2014	South Africa	1044	First and second	0.48 and 0.33	All criteria met
Lyons et al. (2017)	MSM and FSW, mostly aged 25+ years	Prospective cohort study	2013–2016	Senegal	1482	First, second and third	0.13, 0.76 and 0.64	Criteria partially met
MacKellar et al. (2019)	Individuals aged 18–49 years	Prospective longitudinal cohort study	2014–2017	Tanzania	5067	Second	0.23	All criteria met
Mafigiri et al. (2017)	Individuals aged 15–24 years	Cross‐sectional study	2013–2014	Uganda	792	Second	0.07	Criteria partially met
Maina et al. (2020)	Individuals living with HIV on ART, age ≥18 years	Retrospective cohort study	2017–2019	Kenya	549	Third	0.5	All criteria met
Maman et al. (2015)	Individuals aged 15–59 years	Population‐based cross‐sectional study	2012	Kenya	6076	First	0.55	All criteria met
Maman et al. (2016)	Individuals aged 15–59 years	Retrospective nested cohort study	2013	Malawi	7270	First	0.66	All criteria met
Manne‐Goehler et al. (2019)	Individuals age ≥40 years	Prospective longitudinal cohort study	2014–2015	South Africa	4560	Second	0.47	Criteria partially met
Marinda et al. (2020)	Individuals age ≥15 years	Population‐based cross‐sectional survey study	2017–2018	South Africa	36,627	First, second and third	0.78, 0.68 and 0.82	Criteria partially met
Mbengue et al. (2019)	Individuals living with HIV on ART, age ≥18 years	Prospective cohort study	2012–2014	South Africa	353	Third	0.53	All criteria met
Mekuria et al. (2016)	Individuals living with HIV on ART, mean age 37.7 years (SD 9.3 years)	Prospective cohort study	2009–2013	Ethiopia	642	Third	0.92	All criteria met
Merrill et al. (2020)	Individuals living with HIV on ART, age 15–24 years	Cross‐sectional study	2017–2018	Zambia	272	Third	0.59	Criteria partially met
Meshesha et al. (2020)	Individuals living with HIV on first‐line ART, age of cases and controls was 31.6 years (SD ±10.72) and 36.6 years (SD±9.48), respectively	Unmatched case–control study	2016–2018	Ethiopia	389	Third	0.66	All criteria met
Mogosetsi et al. (2018)	Individuals living with HIV on ART, age ≥21 years	Prospective cohort study	2012–2013	South Africa	98	Third	0.97	All criteria met
Mokhele et al. (2019)	Individuals living with HIV on ART, age ≥18 years	Retrospective cohort study	2004–2014	South Africa	3685	Third	0.53	Criteria partially met
Moyo et al. (2016)	Individuals living with HIV on ART, age ≥18 years	Retrospective cohort study	2007–2012	South Africa	13,475	Third	0.91	All criteria met
Mshweshwe‐Pakela et al. (2020)	Individuals living with HIV, mostly age ≥30 years	Retrospective clinical review	2017	South Africa	826	Third	0.77	Criteria partially met
Muraguri et al. (2015)	MSM, age ≥18 years	Cross‐sectional study	2010	Kenya	563	First	0.34	Criteria partially met
Nakanyala et al. (2016)	Individuals, age ≥15 years	Cross‐sectional study	2014–2015	Namibia	2163	First, second and third	0.41, 0.31 and 0.79	Criteria partially met
Ndagijimana et al. (2019)	Individuals living with HIV on ART, median age 34 years (IQR 27–41 years)	Retrospective cohort study	2012–2015	Rwanda	775	Third	0.82	All criteria met
Nega et al. (2020)	Individuals living with HIV on first‐line ART, age ≥10 years	Hospital‐based cross‐sectional study	2018–2019	Ethiopia	295	Third	0.83	All criteria met
Negash et al. (2020)	Individuals living with HIV on ART, age 5–78 years	Hospital‐based cross‐sectional study	2019	Ethiopia	393	Third	0.85	Criteria partially met
Ng'ang'a et al. (2014)	Individuals aged 15–64 years	Population‐based cross‐sectional study	2012–2013	Kenya	13,720	First	0.38	All criteria met
Nnambalirwa et al. (2016)	Individuals living with HIV on first‐line ART, median age 36.7 years (IQR 31.5–43.3 years)	Retrospective cohort study	2004–2011	South Africa	11,724	Third	0.84	All criteria met
Novitsky et al. (2015)	Individuals aged 16–64 years	Community‐based open prospective cohort study	2010–2013	Botswana	6238	First	0.66	Criteria partially met
Novitsky et al. (2018)	Individuals living with HIV aged 16–29 years	Population‐based cross‐sectional study	2013–2015	Botswana	552	First, second and third	0.87, 0.90 and 0.95	Criteria partially met
Nsanzimana et al. (2019)	Individuals living with HIV on second‐line ART, median age 41 years (IQR 33–49 years)	Retrospective observational cohort study	2004–2016	Rwanda	1688	Third	0.79	All criteria met
Nuwagaba‐Biribonwoha et al. (2019)	Individuals living with HIV, age ≥18 years	Cross‐sectional study	2013–2014	South Africa	2196	First	0.09	Criteria partially met
Omooja et al. (2019)	Individuals living with HIV on ART, median age 36 years (IQR 30–44 years)	Cross‐sectional study	2016–2017	Uganda	1169	Third	0.91	Criteria partially met
Ondoa et al. (2020)	Individuals living with HIV on ART, mostly age ≥25 years	Prospective cohort study	2008–2015	Kenya, South Africa, Zambia, Nigeria, Zimbabwe and Uganda	2420	Third	0.87	Criteria partially met
Petersen et al. (2017)	Individuals living with HIV on ART, age ≥15 years	Cross‐sectional study	2013–2014 to 2015–2016	Kenya and Uganda	77,774	Third	0.87	All criteria met
Pulerwitz et al. (2019)	Individuals aged 18–49 years	Population‐based cross‐sectional survey study	2014	South Africa	2019	Second	0.79	All criteria met
Ramadhani et al. (2018)	MSM, age ≥16 years	Community‐based prospective cohort study	2013–2017	Nigeria	1506	First, second and third	0.58, 0.37 and 0.71	Criteria partially met
Reynolds et al. (2018)	Men aged 20–34 years	Cross‐sectional study	2016–2017	Swaziland	568	Second	0.96	All criteria met
Rhead et al. (2019)	Men aged 15–54 years	Cross‐sectional survey study	2012–2013	Zimbabwe	3116	First and second	0.47 and 0.77	Criteria partially met
Riedel et al. (2018)	Individuals living with HIV on ART, age 14–86 years	Retrospective cohort study	2008–2010	Rwanda	531	Third	0.93	All criteria met
Rohr et al. (2017)	Individuals age ≥40 years	Open cohort general‐population survey study	2014–2015	South Africa	4560	First	0.55	All criteria met
Ross et al. (2020)	Individuals living with HIV on ART, age ≥15 years	Cross‐sectional study	2018	Rwanda	12,238	Third	0.91	All criteria met
Shanaube et al. (2017)	Individuals age ≥18 years	Community‐randomized trial	2013–2015	Zambia	101,102	First	0.59	All criteria met
Ssemwanga et al. (2020)	Individuals living with HIV on first‐line ART	Clinic‐based cross‐sectional survey study	2017	Uganda	1611	Third	0.93	All criteria met
Steiner et al. (2020)	Individuals age 18–49 years	Population‐based pre‐post cross‐sectional survey study	2013–2014	Tanzania	5067	Second	0.22	All criteria met
Thin et al. (2019)	Individuals age 15–59 years	Household‐based cross‐sectional survey study	2016–2017	Lesotho	11,682	First, second and third	0.77, 0.77 and 0.92	Criteria partially met
Twahirwa Rwema et al. (2020)	MSM and TGW age ≥ 18 years	Cross‐sectional bio‐behavioural survey study	2018	Rwanda	736	First, second and third	0.61, 0.98 and 0.75	All criteria met
Umar et al. (2019)	Adolescents and young adults living with HIV on ART, aged 13–24 years	Cross‐sectional study	2016	Malawi	209	Third	0.53	All criteria met
Villa et al. (2020)	Individuals living with HIV on ART, median age 48 years (IQR 42–54 years)	Prospective cohort study	2018	Ghana	333	Third	0.39	All criteria met
Wafula et al. (2014)	Individuals living with HIV, age 15–64 years	Population‐based household cross‐sectional survey study	2012–2013	Kenya	363	Second	0.67	All criteria met
Wirth et al. (2020)	Individuals age 16–64 years	Prospective longitudinal cohort study	2013–2018	Botswana	10,791	First, second and third	0.78, 0.70 and 0.96	Criteria partially met
Young et al. (2020)	Individuals living with HIV, age 15–64 years	Household‐based cross‐sectional survey study	2012	Kenya	648	First and second	0.44 and 0.85	Criteria partially met

### A.4 TABLE OF META‐SYNTHESIS CONSTRUCTS

**Table A3:** Constructs from meta‐synthesis of qualitative studies on men's engagement in HIV care

		Third‐order constructs	Second‐order constructs	Summary definition	First‐order constructs	Source(s)
Perceived social norms	*Barriers*	Femininity of healthcare and HIV	Health facilities as feminine spaces	Perception that healthcare facilities are primarily oriented towards addressing the needs of women and children. Also, the staff at clinics are mostly women, which makes it harder for men to feel comfortable discussing their concerns.	*It is difficult because the other problem is that virtually all the nurses and counsellors at the clinic are women and thus men are not comfortable discussing their issues with women. We men prefer talking to other men if we have health problems and thus it is hard to go to the clinic for help*. [Fleming, p. 7]	Adams 2017, Adeagbo 2019, Camlin 2016, Chikovore 2016, Fleming 2016, Lavender 2019, Mak 2016, Martínez Pérez 2016, Mburu 2014, Orr 2017, Osingada 2019, Rankin‐Williams 2017, Tibbels 2019, Zissette 2016
			HIV as a feminine issue	Perception that women are responsible for “managing” HIV in a relationship. This includes testing so that men can know their status by proxy, which, therefore, makes it unnecessary for men to test if their female partner already has tested.	*What [men] like to say is that once I have tested, he had already tested too. (Woman in focus group)* [DiCarlo, p. 15]	Camlin 2016, DiCarlo 2014, Lavender 2019, Mak 2016
		HIV as a threat to social norms	Health, strength and sexuality	Men fear being seen as weak if seen involved in HIV care. They also fear that HIV and HIV treatment could lead to sexual dysfunction and/or take away from a strong and attractive physical appearance.	*From the culture, [to be a man] means to be strong, to have a family. To have your things. Cattles. To get a house. The problem about this, it's never been discussed health issues about men. The only health issue they know is going to the bush [circumcision ceremony] and they come out as man… That they are HIV, it's still a taboo, they hear it in the radio, they see in the TV…* [Martínez Pérez, p. 6]	Adams 2017, Chikovore 2016, DiCarlo 2014, Fleming 2016, Hendrickson 2019, Jennings 2017, Mak 2016, Martínez Pérez 2016, Mooney 2017, Naugle 2019, Ndyabakira 2019, Okal 2020, Orr 2017, Osingada 2019, Rankin‐Williams 2017, Russell 2019, Sileo 2019b, Skovdal 2019, Wamoyi 2017
			Livelihood	Belief that HIV as well as engaging in HIV care takes away from men's ability to earn a livelihood and support their family.	*I had spent a long time without testing because I am always busy looking for money so one would not get time even to go to [nearest health center] get tested*. [Ndyabakira, p. 3]	Chikovore 2016, Naugle 2019, Ndyabakira 2019
			Social standing	Fear that HIV and being seen engaging in HIV care will take away from a man's social and family standing.	*When you suffer from some of these illnesses [HIV], you find that you do not spend enough time with other men as you have to constantly go to the hospital. Your absence from other men may make you feel less masculine… (South Africa, Age 62)* [Fleming, p. 7]	Adams 2017, Camlin 2016, Chikovore 2016, DiCarlo 2014, Fleming 2016, Lavender 2019, Mantell 2019, Martínez Pérez 2016, Mburu 2014, Naugle 2019, Russell 2019, Sileo 2019b, Wamoyi 2017, Zissette 2016
			Lifestyle	Fear that HIV and engagement in HIV care will make life less fun for men because of HIV stigma in the community and the need to take treatment.	*For us, men, HIV is the end of your fun, the end of your joy… it's like you are condemned. When you do not have AIDS, you go to bars, you drink your beer, you find a girl, you go and enjoy…But once you have it, in your neighborhood, if people say you have it, they will point fingers at you. (Man in focus group, age 25–34)* [Naugle, p. 6]	Chikovore 2016, Conserve 2019, DiCarlo 2014, Hendrickson 2019, Naugle 2019, Ndyabakira 2019, Orr 2017, Rankin‐Williams 2017, Sileo 2019b, Zissette 2016
	*Facilitators*	Coping skills	Appearance of health and strength	Belief that engaging in HIV care is a way to maintain and enhance physical appearance, health, strength and sexuality.	*In the past there was so much fear [about HIV] …[but now] I drink my beer and I tell the people around that I am HIV infected, and I am proud…I show off because I look good. (Man with HIV)* [Russell, p. 1203]	Brown 2019, Camlin 2016, Graham 2018, Hendrickson 2019, Russell 2019, Sandfort 2015, Sileo 2019b
			Responsibility for family	HIV can serve as an impetus to help men realize the importance of taking care of themselves so that they are able to take care of their family. Men also described testing in order to start a serious relationship or get married.	*My children are a reason to fight for my life so I can take care of them…[before HIV] I did not know how to save money or even budget, and used [money] for things that did not matter, but ever since I was told that I am HIV positive, I realized that I had to plan… (Man with HIV)* [Russell, p. 1204]	Brown 2019, Camlin 2016, Hendrickson 2019, Mak 2016, Okal 2020, Russell 2019, Sandfort 2015, Schatz 2018, Sileo 2019a, Sileo 2019b, Wamoyi 2017
			Power over HIV	A sense of control over HIV and a desire to fight the diagnosis by engaging in care.	*They gave me ARVs as treatment and therefore I have no reason to be afraid. When someone gives you an instrument like a shield to fight with in a war, do you say that I am afraid? You have to fight. (Man with HIV)* [Russell, p. 1207]	Hendrickson 2019, Osingada 2019, Russell 2019, Sileo 2019a, Sileo 2019b
			New social support	Men with HIV serve as friends and role models for other men with HIV, helping them to see how HIV and masculinity can be compatible. Men also benefit from support from other friends, family and community members.	*Before being diagnosed with HIV, I used to fall sick all the time, yet I know of friends who have already initiated on ART therapy. So, my friends would advise me that why don't you go to a health facility such that you can be checked. (Fisherman, age 23)* [Sileo, p. 781]	Daniels 2019, Graham 2018, Hill 2018, Mburu 2014, Mooney 2017, Osingada 2019, Rosen 2020, Russell 2019, Sileo 2019b, Wamoyi 2017, Zissette 2016
Health system challenges	*Barriers*	Low‐resourced clinics	Lack of materials, medications and/or staff	Frustration that health facilities have long waiting times, unavailable providers, and unavailable testing kits and/or ART.	*When I come, [the provider gives me advice, he tells me to take my medication. I tell him yes I will take the medication, but often when I come there is no medication. So when there's no medication like that, I am discouraged*. [Tibbels, p. 8]	Adams 2017, Adeabgo 2019, Daniels 2019, Krakowiak 2020, Lavender 2019, Mak 2016, Ndyabakira 2019, Ogunbajo 2018, Okal 2020, Tibbels 2019, Tsang 2019, Zissette 2016
		Mistrust and misinformation about HIV	Doubt accuracy of test results	Mistrust that testing results are valid, as well as the ability or motivation of providers to correctly interpret and communicate results.	*I do not doubt the reliability of the test, the test we all know that it is reliable, but sometimes the person who does the test can be wrong. (Man in focus group, age 35–49)* [Tibbels, p. 6]	Graham 2018, Jennings 2017, Martínez Pérez 2016, Ogunbajo 2018, Okal 2020, Osingada 2019, Sandfort 2015, Tibbels 2019
			Misinformation about HIV	False beliefs including that it is impossible to survive with HIV, that HIV is man‐made or that one's personal risk is low despite high‐risk activities.	*Some [men] get involved [in HIV care] while others fear because they think if I am found positive, I would die quickly, so they better go when they are already bedridden*. [Ndyabakira, p. 6]	Adams 2017, Camlin 2016, DiCarlo 2014, Jennings 2017, Mak 2016, Martínez Pérez 2016, Mooney 2017, Ndyabakira 2019, Ogunbajo 2018, Rankin‐Williams 2017, Russell 2019
		Unwanted disclosure	Lack of confidentiality given clinic layout and procedures	Concern that the lack of confidential spaces and procedures within the clinical setting leads to unwanted disclosure.	*… At the hospital there is a bench for those with HIV. When you sit there, you wait for medication, people know that you have HIV. The person has so much fear of this, so much shame, that the person will not go there*. [Tibbels, p. 7]	Adams 2017, Adeagbo 2019, Fleming 2016, Hendrickson 2019, Mak 2016, Mantell 2019, Martínez Pérez 2016, Ogunbajo 2018, Okal 2020, Orr 2017, Rankin‐Williams 2017, Rosen 2020, Sandfort 2015, Tibbels 2019, Van Heerden 2015, Zissette 2016
		Anticipated and enacted stigma	Enacted and anticipated stigma towards persons with HIV	Experienced of expected judgement from healthcare staff and others towards people with HIV.	*When you get to the hospital, you feel as if you have failed, being sick…When they discover it is HIV, they give you a weird look. When your back is turned, the staff laughs…. I lived it yesterday and it hurt me. (Man with HIV, age 25–34)* [Tibbels, p. 5]	Adams 2017, Adeagbo 2019, Chikovore 2016, Daniels 2019, DiCarlo 2014, Hendrickson 2019, Mak 2016, Mantell 2019, Micheni 2017, Mooney 2017, Naugle 2019, Ndyabakira 2019, Ogunbajo 2018, Okal 2020, Orr 2017, Osingada 2019, Rankin‐Williams 2017, Rosen 2020, Russell 2019, Sandfort 2015, Skovdal 2019, Tibbels 2019, Tsang 2019, Zissette 2016
			Enacted and anticipated stigma towards MSM, regardless of HIV status	Experienced or expected judgement from healthcare staff and others towards MSM, which intersects with stigma of HIV	*If I went to a health facility the moment I meet you I can tell how homophobic you are or how friendly you are … I cannot access health care where there is stigma or a place where they are not sensitive to sexuality issues. (MSM age 22 years, ART‐naïve)* [Graham, p. 100]	Daniels 2019, Graham 2018, Mak 2016, Micheni 2017, Sandfort 2015, Tsang 2019
	*Facilitators*	Convenient access to healthcare	Home testing	Home testing allows for a comfortable, private environment for testing and is enhanced by health counsellors.	*I think men definitely would [home]‐test … because there is no place like home. It is where I know I can get all the support*. [DiCarlo, p. 877]	DiCarlo 2014, Krakowaik 2020, Martínez Pérez 2016, Ndyabakira 2019, Rankin‐Williams 2017, Van Heerden 2015 [118,125,131,139,190,193, 127,134,140,148,199,202, 128,135,141,149,200,203, 125,201,202, 127,134,140,148,199,202, 126,133,139,147,198,201, 124,131,137,145,196,199, 122,129,135,143,194,197, 121,128,134,142,193,196, 119,126,132,140,191,194, 120,127,133,141,192,195, 119,126,132,140,191,194, 118,125,131,139,190,193, 115,122,128,136,187,190, 113,120,126,134,185,188, 112,119,125,133,184,187, 111,118,124,132,183,186]
			Self‐testing	Self‐testing gives men a sense of control, privacy and convenience.	*Yeah, like none sees me while I test. And once I am done, I throw it to the dustbin. (Man, age 26)* [Jennings, p. 5]	Adeagbo 2019, Jennings 2017, Osingada 2019
			Flexible clinic opening hours	Flexible facility hours help to accommodate busy work schedules.	*…they should make these hospitals operate 24 hours because this one is a big hospital…. The reason why I'm saying that is because there are some people who work in daytime up to very late and when they come here they don't get services because the doctors and the nurses are gone. (Man with HIV, age 40)* [Okal, p. 14]	Okal 2020
		Trust in health system	Belief in effectiveness of ART	Belief in ART effectiveness through clinical guidance, public advertisements and personal experience.	*So I believe that this treatment is good because my skin was black, and I also lost weight, but I have recovered. People, they used to ask me what I am eating nowadays because I look healthy and my body has recovered, compared to last year. So I have started to realize that ART is helpful, and it's true. (Man with HIV, age 45–50)* [Mooney, p. 279]	Brown 2019, DiCarlo 2014, Hendrickson 2019, Mooney 2017, Ogunbajo 2018, Okal 2020, Rankin‐Williams 2017, Russell 2019, Schatz 2018, Skovdal 2019
			Positive experiences with healthcare staff	Experiences in which healthcare staff have been especially helpful to men engaging in care.	*When I went to the hospital, I didn't tell my parents and I did not have any money. The nurse that counseled me, she paid for my labs. I needed labs before they could put me on the medicine. The nurse I went to see paid for my labs and she is the one who made everything easier for me*. [Ogunbajo, p. 836]	Brown 2019, Graham 2018, Mak 2016, Ogunbajo 2018
Poverty	*Barriers*	Poverty	Direct unaffordability of seeking HIV care	Economic challenges, including transport costs, non‐subsidized medical expenses and costs for medical visits. The informal health system is perceived as more affordable.	*Think that if they go to the hospital, the costs will be exorbitant. So they prefer to stay in their corners, do their traditional treatment. (Man in focus group, age 25–34)* [Tibbels, p. 8]	Jennings 2017, Mak 2016, Micheni 2017, Ndyabakira 2019, Ogunbajo 2018, Schatz 2018, Sileo 2019b, Tibbels 2019
			Opportunity costs of care	HIV care takes time and money away from needing to seek employment, food and other needs.	*You see the challenge that most of us have faced is that we are poor; the illness finds us in poverty. So, you have to strive hard to look for money and that involves use of a lot of energy, which is a very big challenge*. (Boat operator, age 32) [Sileo, p. 780]	Adeabgo 2019, Camlin 2016, Jennings 2017, Krakowiak 2020, Mak 2016, Micheni 2017, Ndyabakira 2019, Ogunbajo 2018, Sileo 2019a, Tibbels 2019
	*Facilitators*	Affordability of care	Testing alternatives	Home and self‐testing perceived as more affordable because they cost less and they are quicker, so men lose less productive time. Home testing is preferred because men do not have to spend money to travel to clinics or hospitals.	*The advantage [of HIV self‐testing kits] is there is no need to go to the hospital to take the test. And also, you save money because…in private hospitals you must pay to be tested. (Man, age 22)* [Jennings, p. 5]	Adeagbo 2019, Camlin 2016, Jennings 2017, Krakowiak 2020, Mak 2016, Micheni 2017, Ndyabakira 2019, Ogunbajo 2018, Sileo 2019a, Tibbels 2019

### A.5 FOREST PLOTS COMPARING MEN AND WOMEN FOR EACH OF THE UNAIDS 95‐95‐95 GOALS

Figures A1, A2, A3, A4, A5, A6, A7, A8, A9, A10, A11, A12, A13, A14, A15, A16
